# Gut microbiota from high-risk men who have sex with men drive immune activation in gnotobiotic mice and *in vitro* HIV infection

**DOI:** 10.1371/journal.ppat.1007611

**Published:** 2019-04-04

**Authors:** Sam X. Li, Sharon Sen, Jennifer M. Schneider, Ka-Na Xiong, Nichole M. Nusbacher, Nancy Moreno-Huizar, Michael Shaffer, Abigail J. S. Armstrong, Erin Severs, Kristine Kuhn, Charles P. Neff, Martin McCarter, Thomas Campbell, Catherine A. Lozupone, Brent E. Palmer

**Affiliations:** 1 Division of Allergy and Clinical Immunology, Department of Medicine, University of Colorado Anschutz, Aurora, Colorado, United States of America; 2 Division of Biomedical Informatics and Personalized Medicine, Department of Medicine, University of Colorado Anschutz, Aurora, Colorado, United States of America; 3 Department of Immunology and Microbiology, University of Colorado Anschutz, Aurora, Colorado, United States of America; 4 Division of Rheumatology, Department of Medicine, University of Colorado Anschutz, Aurora, Colorado, United States of America; 5 Division of Colorectal Surgery, Department of Medicine, University of Colorado Anschutz, Aurora, Colorado, United States of America; 6 Division of Infectious Diseases, University of Colorado Anschutz, Aurora, Colorado, United States of America; Emory University, UNITED STATES

## Abstract

Men who have sex with men (MSM) have differences in immune activation and gut microbiome composition compared with men who have sex with women (MSW), even in the absence of HIV infection. Gut microbiome differences associated with HIV itself when controlling for MSM, as assessed by 16S rRNA sequencing, are relatively subtle. Understanding whether gut microbiome composition impacts immune activation in HIV-negative and HIV-positive MSM has important implications since immune activation has been associated with HIV acquisition risk and disease progression. To investigate the effects of MSM and HIV-associated gut microbiota on immune activation, we transplanted feces from HIV-negative MSW, HIV-negative MSM, and HIV-positive untreated MSM to gnotobiotic mice. Following transplant, 16S rRNA gene sequencing determined that the microbiomes of MSM and MSW maintained distinct compositions in mice and that specific microbial differences between MSM and MSW were replicated. Immunologically, HIV-negative MSM donors had higher frequencies of blood CD38+ HLADR+ and CD103+ T cells and their fecal recipients had higher frequencies of gut CD69+ and CD103+ T cells, compared with HIV-negative MSW donors and recipients, respectively. Significant microbiome differences were not detected between HIV-negative and HIV-positive MSM in this small donor cohort, and immune differences between their recipients were trending but not statistically significant. A larger donor cohort may therefore be needed to detect immune-modulating microbes associated with HIV. To investigate whether our findings in mice could have implications for HIV replication, we infected primary human lamina propria cells stimulated with isolated fecal microbiota, and found that microbiota from MSM stimulated higher frequencies of HIV-infected cells than microbiota from MSW. Finally, we identified several microbes that correlated with immune readouts in both fecal recipients and donors, and with *in vitro* HIV infection, which suggests a role for gut microbiota in immune activation and potentially HIV acquisition in MSM.

## Author summary

The communities of commensal microbes that colonize the human gut comprise the gut microbiome, which has been shown to play a significant role in shaping the immune system. Recent studies have reported a distinct gut microbiome composition in men who have sex with men (MSM) exhibiting HIV-risk behaviors when compared with low-risk men who have sex with women (MSW), regardless of their HIV infection status. Whether these gut microbiome differences in high-risk MSM directly impact immune activation is important to understand since increased T cell activation is associated with increased HIV transmission risk and more severe disease. To test the immunological effect of the gut microbiome in MSM, we transplanted stool from HIV-negative MSW, HIV-negative high-risk MSM, and HIV-positive MSM to germ-free mice. DNA sequencing showed that specific microbiome differences associated with MSM were successfully engrafted in mice, and that these differences were associated with increased CD4+ and CD8+ T cell activation in the mice. These results provide evidence for a direct link between microbiome composition and immune activation in HIV-negative and HIV-positive MSM, and rationale for investigating the gut microbiome as a risk factor for HIV transmission.

## Introduction

Men who have sex with men (MSM) comprise over half of all people living with HIV in the United States and accounted for 67% of new U.S. infections in 2016 [1]. Prevention and treatment in the MSM population is of high priority in the effort to eradicate HIV/AIDS in the U.S. Identifying novel biological factors that potentially impact transmission and/or disease in MSM could open opportunities for unique prevention and treatment strategies.

Recent studies have found that a distinct gut microbiome composition is found in MSM when compared with heterosexual men (men who have sex with women, MSW) even in the absence of HIV infection [2–4]. Interestingly, several studies have shown that high-risk HIV-negative MSM also exhibit immune differences, such as higher blood T cell activation [5], increased endotoxemia [6], and increased T cell pro-inflammatory cytokine production in colon mucosa [3], when compared with HIV-negative MSW. Given that the gut microbiome plays a significant role in shaping the immune system [7], it is possible that the gut microbiome differences observed in HIV-negative MSM may be a driving factor of increased immune activation in this population. Subtler microbiome differences have also been linked with chronic HIV infection itself when controlling for MSM [2, 4, 8], and previous work from our group has shown that these subtle differences are associated with stronger stimulation of immune cells *in vitro* [8]. How HIV-associated microbiome differences impact immune activation *in vivo* remains unknown. Establishing a direct impact of the gut microbiome on immune activation in HIV-negative and HIV-positive MSM may have important implications for transmission and disease, since vaginal immune activation in women has been associated with HIV acquisition risk [9–12] and T cell activation is well known to be a correlate of disease progression in HIV-positive individuals [13]. Whether the gut microbiome may be a risk factor for HIV transmission in MSM has not been investigated.

Direct causation of immune activation by the microbiome in HIV-negative and HIV-positive MSM is difficult to demonstrate with human studies, which can be limited to correlational analyses to establish microbial-immune relationships. Human studies can also be confounded by factors such as diet, age, and lifestyle behaviors, all of which can be variable across individuals and populations and may influence both the microbiome and the immune system [14–17]. Deciphering microbiota-associated immune effects in HIV-positive MSM is further complicated by HIV itself, which can cause depletion of critical microbiome-interacting T cells [18, 19] and stimulate immune activation through TLRs [20]. These challenges raise the need for an *in vivo* model to elucidate the immunological impacts of gut microbiota from HIV-negative and HIV-positive MSM. Mice have been crucial for describing the roles of gut microbiota in health and disease [21], and offer a controlled system to isolate effects of live, active microbes on the immune system while excluding effects of lifestyle and HIV itself. In this study, we leveraged gnotobiotic (germ-free) mice to investigate the immunological impacts of whole fecal transplantation from HIV-negative MSW, HIV-negative MSM, and HIV-positive antiretroviral therapy (ART)-naïve MSM. We report that mouse fecal recipients recapitulated some of the microbiome differences associated with MSM, and that mouse recipients of feces from HIV-negative and HIV-positive MSM exhibited higher gut T cell activation than recipients of HIV-negative MSW. We extended our findings from the mouse model to an *in vitro* HIV infection model, and found that stimulation of primary human lamina propria cells (LPCs) with isolated fecal microbiota from either HIV-negative or HIV-positive MSM promoted HIV infection in cell culture. These results demonstrate the connection between gut microbiota and immune phenotypes observed in MSM, which may have implications for HIV acquisition and disease.

## Results

### T cell phenotypes in peripheral blood associated with MSM and HIV

**Fig 1:**
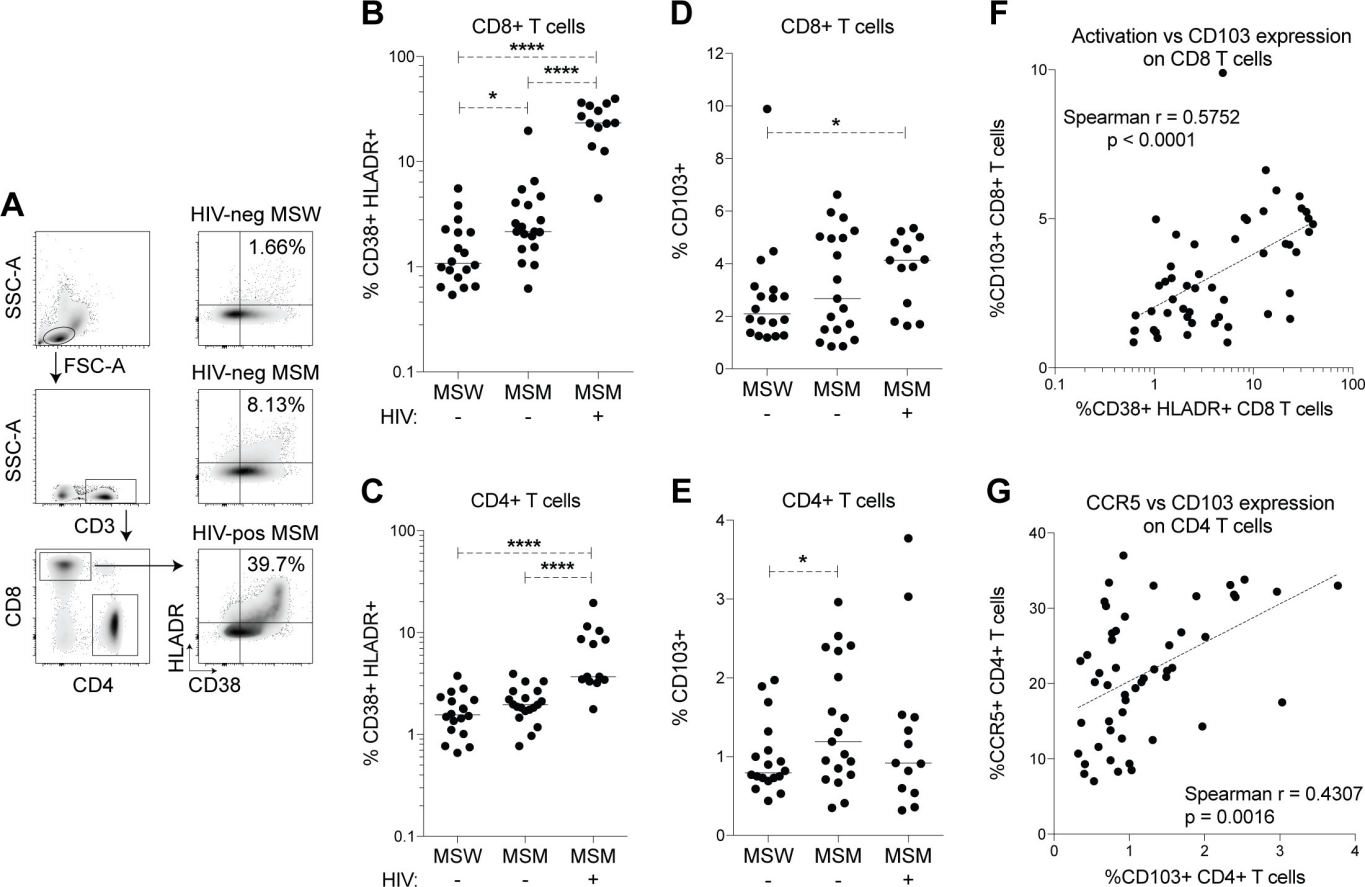
HIV-negative and HIV-positive untreated MSM have higher frequencies of activated and mucosal homing T cells in the blood. PBMC from HIV-negative MSW (n = 18), HIV-negative MSM (n = 19), and HIV-positive MSM (n = 13) were stained for T cell activation and mucosal homing markers and analyzed by flow cytometry. (A) T cell activation gating strategy. (B–E) Comparison of frequencies of CD38+ HLADR+ (B) CD8+ and (C) CD4+ T cells, and frequencies of CD103+ (D) CD8+ and (E) CD4+ T cells between HIV-negative MSW, HIV-negative MSM, and HIV-positive MSM. Each data point represents an individual, and lines represent median values. Statistical analyses were performed using t-tests to compare groups if data from both groups had normal distributions and Mann-Whitney tests to compare groups if data from at least one group had a non-parametric distribution. **** = p <0.0001, *** = p<0.001, ** = p<0.01, * = p<0.05. (F–G) Spearman correlations between (F) CD8+ T cell activation, plotted on a log scale axis but correlated using linear data, and CD103+ CD8+ T cell frequency, and between (G) CD103+ CD4+ T cell frequency and CCR5+ CD4+ T cell frequency. Dotted lines represent non-linear semi-log (F) and linear (G) regression analysis.

Previous studies have reported higher blood CD4+ and CD8+ T cell activation and higher levels of inflammatory cytokine producing colonic CD8+ T cells in HIV-negative MSM compared with HIV-negative MSW [3, 5, 6]. To confirm these findings in an independent cohort, we analyzed blood T cell phenotypes in 18 HIV-negative MSW and 19 HIV-negative MSM, and included 13 HIV-positive untreated MSM as controls since immune activation is well known to be increased with HIV infection [13, 22, 23] (S1A Table). Increased frequencies of CD38 HLADR expressing CD8+ T cells, but not CD4+ T cells, were found in the blood of HIV-negative MSM compared with HIV-negative MSW (Fig 1A–1C). As expected, HIV-positive MSM had the highest levels of blood T cell activation. Expression of CD69 on peripheral blood T cells was also measured (S1A and S1B Fig), and frequencies of CD69+ CD4+ T cells trended higher for HIV-negative MSM and HIV-positive MSM than HIV-negative MSW, though this did not reach statistical significance. Stimulated T cells were analyzed for intracellular TNF-α and IFN-γ expression, and significantly higher frequencies of inflammatory cytokine producing T cells were observed in HIV-positive, but not HIV-negative, MSM when compared with HIV-negative MSW (S1C and S1D Fig). Finally, a lower CD4/CD8 T cell ratio in the blood has been previously reported for high-risk HIV-negative MSM [6], but was not reproduced in our cohort (S1F Fig). The CD4/CD8 T cell ratio was expectedly decreased in HIV-positive MSM who were not receiving antiretroviral treatment.

Gut mucosal T cell activation plays an important role in HIV replication and disease [24]. We therefore evaluated expression of the mucosal homing marker CD103 (also known as integrin αEβ7) [25] on blood T cells. Frequencies of CD103+ CD4+ T cells were significantly higher and frequencies of CD103+ CD8+ T cells trended higher in HIV-negative MSM compared with HIV-negative MSW (Fig 1D and 1E). CD103+ CD8+ T cell, but not CD4+ T cell frequencies were higher in HIV-positive MSM than in HIV-negative MSW. No significant differences were observed in frequencies of CD103+ T cells between HIV-negative and HIV-positive MSM. Frequencies of CD103+ CD8+ T cells significantly correlated with frequencies of CD38+ HLADR+ CD8+ T cells (Fig 1F), while expression of HIV coreceptor CCR5 [26]–which was not significantly different across groups (S1E Fig)–significantly correlated with frequencies of CD103+ CD4+ T cells (Fig 1G). Taken together, previous findings of higher immune activation in HIV-negative MSM compared with HIV-negative MSW are confirmed here in our cohort, and our results additionally show an association between blood T cell activation and mucosal homing in MSW and MSM.

### Microbiome differences between MSW and MSM are transferred to mice through fecal transplant

**Fig 2:**
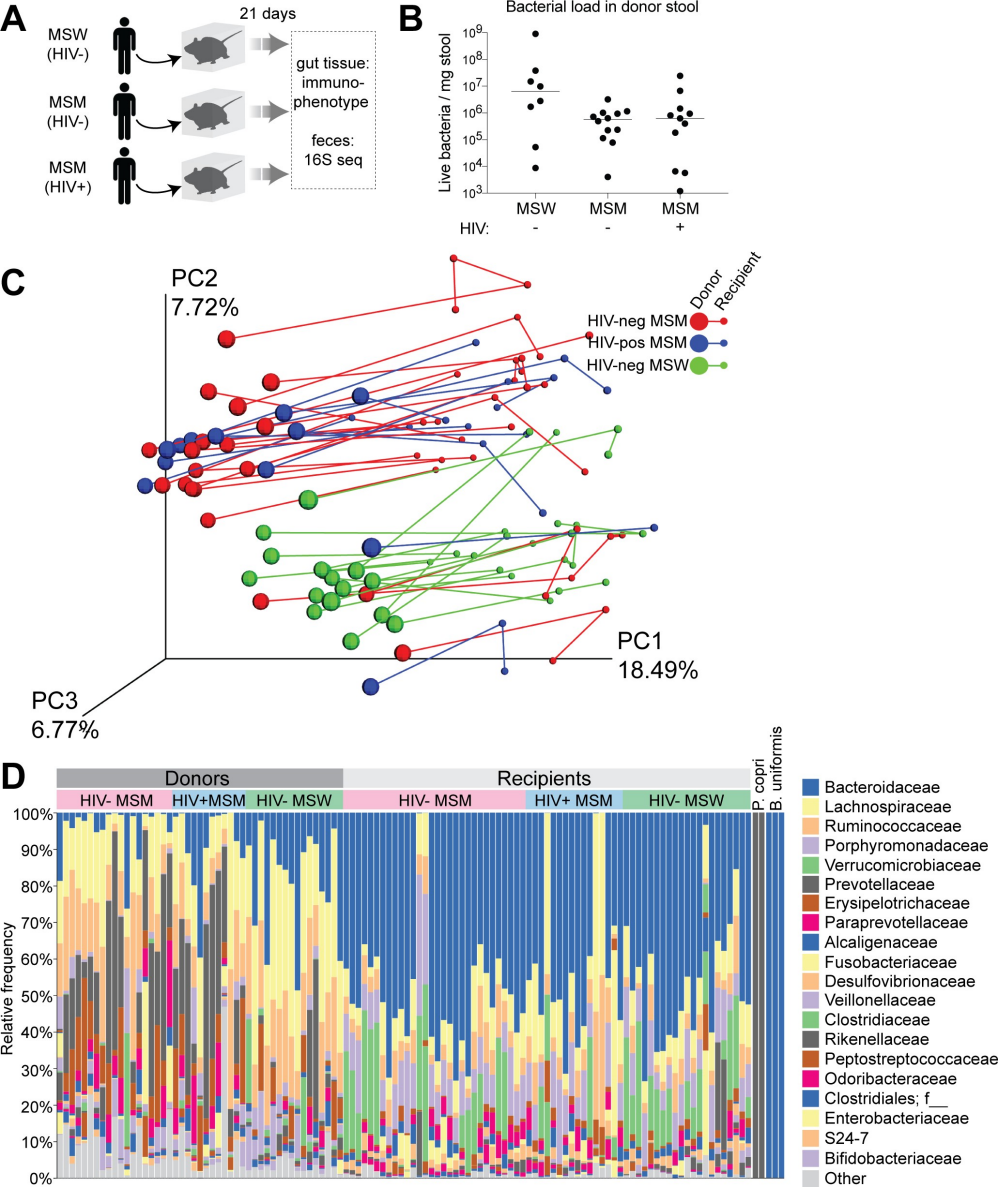
Microbiome composition differences between MSW and MSM are transferred to mouse recipients by fecal transplant. (A) Gnotobiotic mice were gavaged with feces from HIV-negative MSW (n = 16), HIV-negative MSM (n = 19), and HIV-positive MSM (n = 12) donors and sacrificed 21 days post-gavage for immunological analysis and 16S rRNA gene sequencing. (B) Bacterial load in donor stool. Isolated stool bacteria from a subset of donors (8 HIV-negative MSW, 12 HIV-negative MSM, 11 HIV-positive MSM) were stained and counted by flow cytometry. Each data point represents an individual donor, and lines represent medians. Mann-Whitney tests were performed to compare groups, and no statistically significant differences were detected. (C) Unweighted UniFrac clustering of donor (large dots) and recipient (small dots) microbiome compositions. Green dots represent HIV-negative MSW donor/recipient pairs, red dots represent HIV-negative MSM donor/recipient pairs, and blue dots represent HIV-positive MSM donor/recipient pairs. Lines connect donors to recipients and recipient replicates when applicable. (D) Taxa bar chart showing relative abundance of bacterial families in each donor and recipient. Legend identifies the top 20 most abundant families across all samples. *P*. *copri* refers to mice colonized with pure culture of *Prevotella copri*, and *B*. *uniformis* refers to mice colonized with pure culture of *Bacteroides uniformis*. f__ = unclassified bacterial family.

Microbiome differences have been previously associated with MSM and HIV [2–4, 8], but the extent to which these differences drive immune activation *in vivo* is unclear. To directly assess the *in vivo* immunological effect of the gut microbiome from HIV-negative and HIV-positive MSM, we transplanted feces from human donors to gnotobiotic mice. Each mouse received a single gavage of feces from a unique human donor. Stool donors were 16 HIV-negative MSW, 19 HIV-negative MSM, and 12 HIV-positive ART-naïve MSM randomly selected from a previously described cohort [4]. Of these donors, 13 HIV-negative MSW, 17 HIV-negative MSM, and 9 HIV-positive MSM are represented in the above immune data (Fig 1, S1 Table). HIV-positive MSM donors had a median viral load of 101400 copies/ml with a median CD4 T cell count of 574 cells/μl (S1 Table). Each donor was tested in a single mouse recipient, with the exception of 4 HIV-negative MSW, 8 HIV-negative MSM, and 4 HIV-positive MSM which were randomly chosen to be tested in 2–3 replicate mice. A subset of donors was also tested for fecal bacterial load (8 HIV-negative MSW, 12 HIV-negative MSM, 11 HIV-positive MSM), which was not found to be significantly different across donor groups (Fig 2B), indicating that each recipient group did not receive a significantly higher or lower amount of bacteria in the fecal transplant.

We performed 16S rRNA gene sequencing on donor fecal samples and fecal pellets from mouse recipients at 7, 14, and 21 days post-gavage. Since there was little variation in composition of the mice over time (S2 Fig), we focused our analysis on the terminal timepoint. Consistent with previous reports [2, 4, 8], unweighted UniFrac analysis showed that microbiome composition in the human donors (large circles) clustered distinctly by MSM and not HIV status (Fig 2C). Although the overall composition shifted along PC1 following fecal transfer, the distinct clustering of MSW and MSM along the PC2 axis was replicated in the mouse recipients (small circles), which are connected to their respective donors by lines (Fig 2C). Six 16S rRNA sequence variants were identified to be significantly different (with an FDR corrected p-value<0.1) between HIV-negative MSW and HIV-negative MSM recipients, 3 of which increased (*Desulfovibrio sp*., *Holdemanella biformis*, *Howardella ureilytica*) and 3 of which decreased (*Clostridium sp*., *Bacteroides uniformis*, *Flavonifractor sp*.) with MSM (S2 Table). Five of six of these variants were also found to be significantly different between HIV-negative MSW and HIV-negative MSM donors (S2 Table). *Holdemanella biformis* (formerly *Eubacterium biforme*) and *Bacteroides uniformis* have also previously been described to be different with MSM [4, 8]. In contrast, no statistically significant differences (FDR p<0.1) in variant abundance were detected between HIV-negative and HIV-positive MSM donors or recipients, and previously reported bacteria differing with HIV in analyses of larger cohorts [2, 4] were not observed in this smaller donor sample size (S3 Table). These data suggest that MSM-associated microbiome differences are readily transferred to mice through fecal transplant, while transfer of subtler HIV-associated differences may require an increased sample of donors.

The natural gut microbiome in mice is compositionally different from that in humans [27], therefore changes in relative abundance of microbes less or better adapted to the mouse gut were expected to occur following fecal transfer. To characterize microbiome shifts following colonization of mice, we identified bacterial families that significantly increased or decreased in relative abundance from donors to recipients. When analyzing all groups together (HIV-negative MSW, HIV-negative MSM, HIV-positive MSM), 25 families significantly changed (FDR p<0.1) as determined by nonparametric t-test comparing donors to recipients (S4 Table). These consisted of 19 families that decreased and 7 families that increased in relative abundance in recipients compared with donors. When stratifying the analyses by group, differences between donors and recipients for 20/25 families maintained consistent trends within each group, and differences for 11/25 families maintained statistical significance within each group (S4 Table). A striking donor-to-recipient change was the relative abundance of *Prevotellaceae*, which was significantly higher in MSM compared with MSW (Fig 2C) [2–4], and decreased in abundance for all groups following transfer (S4 Table). Taxa bar charts showing donors and recipients grouped separately (Fig 2D), or donors and their respective recipients side-by-side (S3 Fig), demonstrate the overall loss of *Prevotellaceae* in mice. Though *Prevotellaceae* was undetectable in almost all mouse recipients of HIV-negative and HIV-positive MSM donors, it maintained a strong presence in the two mouse recipients of *Prevotellaceae*-rich HIV-negative MSW donors (Fig 2D, S3 Fig). One reason that there may be colonization differences in MSW versus MSM recipients is the presence of different species or strains of *Prevotella*. We thus sought to explore whether different sequence variants within the *Prevotella* genus showed different colonization patterns. In the two mice that *Prevotella* successfully colonized, we identified only three *Prevotella* sequence variants to be present and all were classified as *Prevotella copri* (S4A Fig). Two out of three *P*. *copri* variants were of the highest abundance in MSW donors (S4B Fig), and increased in relative abundance from donors to mice. The third variant was the dominant and most abundant *Prevotella* sequence in both MSW and MSM donors, but only colonized recipients of MSW. Six other *P*. *copri* sequence variants that were present in at least 20% of MSM donors were identified, and only three of these could be found in *Prevotella*-rich MSW. None of these other six sequence variants were detectable in mouse recipients (S4 Fig). To investigate the ability of *Prevotella* to colonize mice in the absence of competing microbes, we gavaged gnotobiotic mice (n = 3) with a monoculture of *P*. *copri* (DSMZ 18205) or with a monoculture *B*. *uniformis* (ATCC 8492) for comparison (Fig 2D). By 16S gene sequence, the *P*. *copri* monoculture was determined to be the same variant as *P*. *copri* 3 found in fecal donors and their recipients. After 21 days of colonization, sequencing showed that *P*. *copri* was still present in feces of 2/3 mice, while *B*. *uniformis* was present in 3/3 mice (Fig 2D). Thus, three *P*. *copri* variants were able to colonize mice, with two of these variants being at a higher relative abundance in *Prevotella*-rich MSW than in MSM. The third variant, which was the dominant variant in both MSW and MSM, could only successfully colonize mice in pure culture or in the context of an MSW microbiome.

Since MSM have more *Prevotella* than MSW, we next investigated whether the inability of *Prevotella* to colonize mouse recipients of MSM resulted in a larger magnitude donor-to-recipient compositional shift for MSM. Unweighted Unifrac distance, a measurement of relative compositional difference, between each donor and their recipient was calculated. For donors with replicate mouse recipients, a representative mouse was randomly selected. Donor-recipient distances were not found to be significantly different across groups, indicating the overall composition of each donor group was altered on average by the same magnitude after transfer (S5A Fig). The percentages of unique sequence variants identified in each donor that were present in their mouse recipient were also not significantly different across groups, indicating equal colonization fidelity (S5B Fig). Despite the loss of taxa that differed with MSM following transfer, differences in specific microbes were recapitulated in mouse recipients to maintain distinct MSW and MSM compositions. Any immunological differences between recipient groups would therefore be associated with these compositional differences.

### Immune activation and barrier integrity in mouse recipients

**Fig 3:**
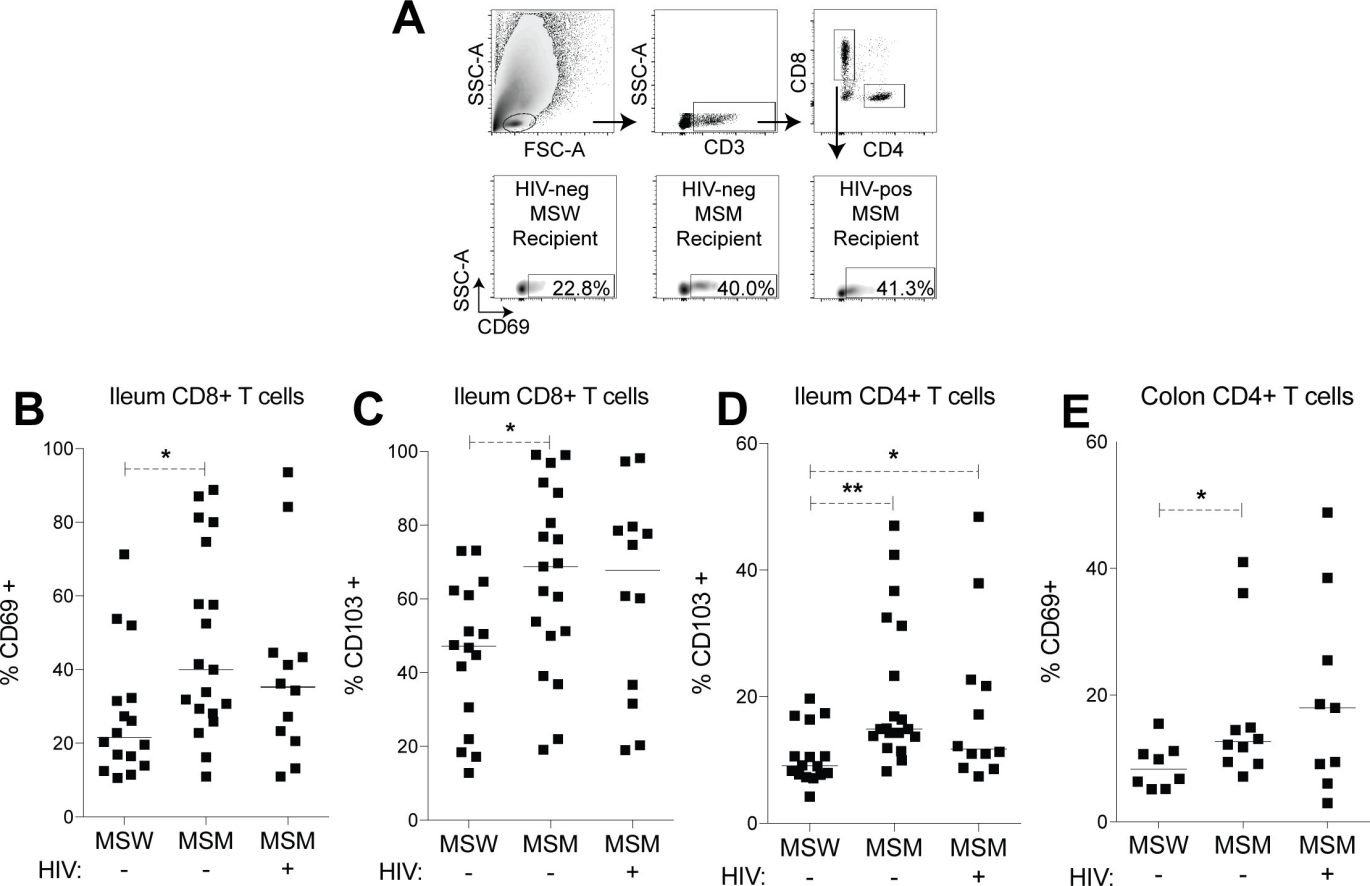
Gut T cell activation and homing in mouse recipients. Ileum and colon tissues were collected from mouse recipients at 21 days post gavage and analyzed for frequencies of CD69+ and CD103+ T cells. (A) Gating strategy for CD69+ T cells. Representative plots from ileum tissues are shown. (B-C) Frequencies of (B) CD69+ CD8+ T cells, (C) CD103+ CD8+ T cells, and (D) CD103+ CD4+ T cells in the ileum. (E) Frequencies of CD69+ CD4+ T cells in the colon. Each data point represents a single mouse gavaged with an individual donor’s feces, and a representative mouse was used for stools tested in replicate mice. Lines represent medians. Statistical analyses were performed using t-tests to compare groups if data from both groups had normal distributions and Mann-Whitney tests to compare groups if data from at least one group had a non-parametric distribution. ** = p<0.01, * = p<0.05.

To investigate the immunological effect of the MSM and HIV-associated microbiome on mouse recipients, mice were sacrificed 21 days post gavage, and ileum, colon, and mesenteric lymph nodes were evaluated for markers of T cell activation. Due to lower cell recovery, only colons from a subset of mice (8 HIV-negative MSW, 10 HIV-negative MSM, 9 HIV-positive MSM) yielded enough cells for analysis by flow cytometry. Ileum tissues from all mice were analyzed and are highly relevant to HIV infection due to the small intestine’s abundance of Th17 cells [28], which are targets of HIV infection and play an important role in disease [18]. CD69 and CD103 were used to measure gut T cell activation, and though lowly expressed in the blood [29], these are relevant markers in the gut, and are used to define tissue-resident memory T cells retained in gut tissue [30, 31]. For stools that were tested in replicate mice, immune data from one replicate was used for each stool. In the ileum, frequencies of CD69+ CD8+ T cells were found to be significantly higher in mouse recipients of HIV-negative MSM compared with mouse recipients of HIV-negative MSW (Fig 3B). Frequencies of CD103+ T cells were significantly elevated for both CD4+ and CD8+ T cells in the ileum of recipients of HIV-negative MSM (Fig 3C and 3D). Interestingly, ileum frequencies of CD69+ or CD103+ T cells were not significantly different between recipients of HIV-negative and HIV-positive MSM, and only CD103+ CD4+ T cells were significantly higher in recipients of HIV-positive MSM compared with recipients of HIV-negative MSW (Fig 3D). Finally, stool recipients that were successfully colonized with *Prevotella* did not display noticeably different levels of immune activation compared to the MSW group median (S7B Fig).

**Fig 4:**
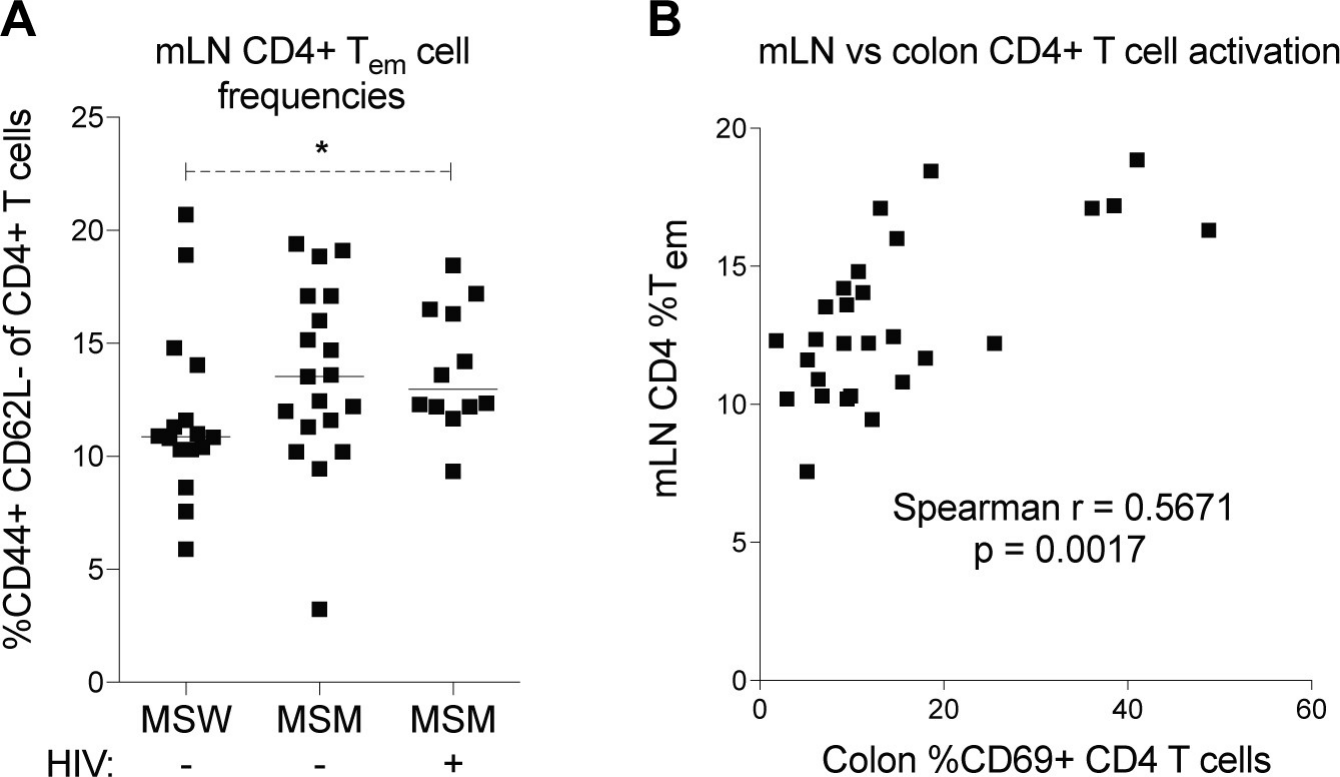
Frequencies of effector memory CD4+ T cells in the mesenteric lymph nodes of mouse recipients. mLN from mouse recipients were collected and analyzed for effector memory CD4+ T cell frequency (CD44+ CD62L-) using flow cytometry. (A) Comparison of frequencies of CD44+ CD62- CD4+ T cells in the mLN across recipient groups. (B) Spearman correlations of effector memory CD4+ T cell (T_em_) frequencies in the mLN with frequencies of CD69+ CD4+ T cells in the colon. Each data point represents a single mouse gavaged with an individual donor’s feces, and a representative mouse was used for stools tested in replicate mice. Lines represent medians. Statistical analyses were performed using t-tests to compare groups if data from both groups had normal distributions and Mann-Whitney tests to compare groups if data from at least one group had a non-parametric distribution. * = p<0.05.

In colon tissue, frequencies of CD69+ CD4+ T cells were significantly higher for recipients of HIV-negative MSM than recipients of HIV-negative MSW (Fig 3E). Recipients of HIV-positive MSM did not statistically differ from recipients of HIV-negative MSM or HIV-negative MSW in colonic T cell activation, but did trend higher for frequencies of CD69+ CD8+ and CD4+ T cells, and CD103+ CD8+ T cells, compared with either HIV-negative group (Fig 3E, S6 Fig). Statistical power in detecting differences in the colon may have been limited by a small sample size and a high variance of immune measures. We therefore examined mesenteric lymph nodes (mLN), which were analyzed for all mouse recipients, as a proxy of gut immune phenotypes since the mLN drain ileum and colon tissues [32]. In the mLN, mouse recipients of HIV-positive MSM displayed significantly higher frequencies of effector memory (T_em_) CD4+ T cells (CD62L-, CD44+) than recipients of HIV-negative MSW (Fig 4A). Furthermore, frequencies of CD4+ T_em_ in the mLN significantly correlated with frequencies of CD69+ CD4+ T cells in the colon (Fig 4B), indicating mLN phenotypes were representative of immune readouts in colon tissue. In mLN of monocolonized mice, which were also examined at 21 days post gavage, CD4+ T_em_ frequencies were within the range detected in stool recipients, and did not differ between mice gavaged with *B*. *uniformis* and mice gavaged with *P*. *copri* (S7A Fig).

Overall, mouse recipients displayed a significant MSM-associated effect in immune activation in the gut. Recipients of MSM also tended to have higher variation in T cell measures, with CD103+ CD4+ T cell frequencies in the ileum (Fig 3D) and CD69+ CD4+ T cell frequencies in the colon (Fig 3E) being significantly different in variance as determined by F test between HIV-negative MSM and HIV-negative MSW. Despite the lack of a significant HIV-associated effect, immunological differences between MSW and MSM recipients were enough to drive significant immune correlations between donors and recipients, including a correlation between frequencies of recipient ileum CD69+ CD8+ T cells and frequencies of donor blood CD38+ HLADR+ CD8+ T cells (S8A Fig), and a correlation between frequencies of recipient colon CD69+ CD4+ T cells and frequencies of HIV-negative donor blood CD103+ CD4+ T cells (S8B Fig). These correlations highlight immunological similarities between donors and their recipients.

**Fig 5:**
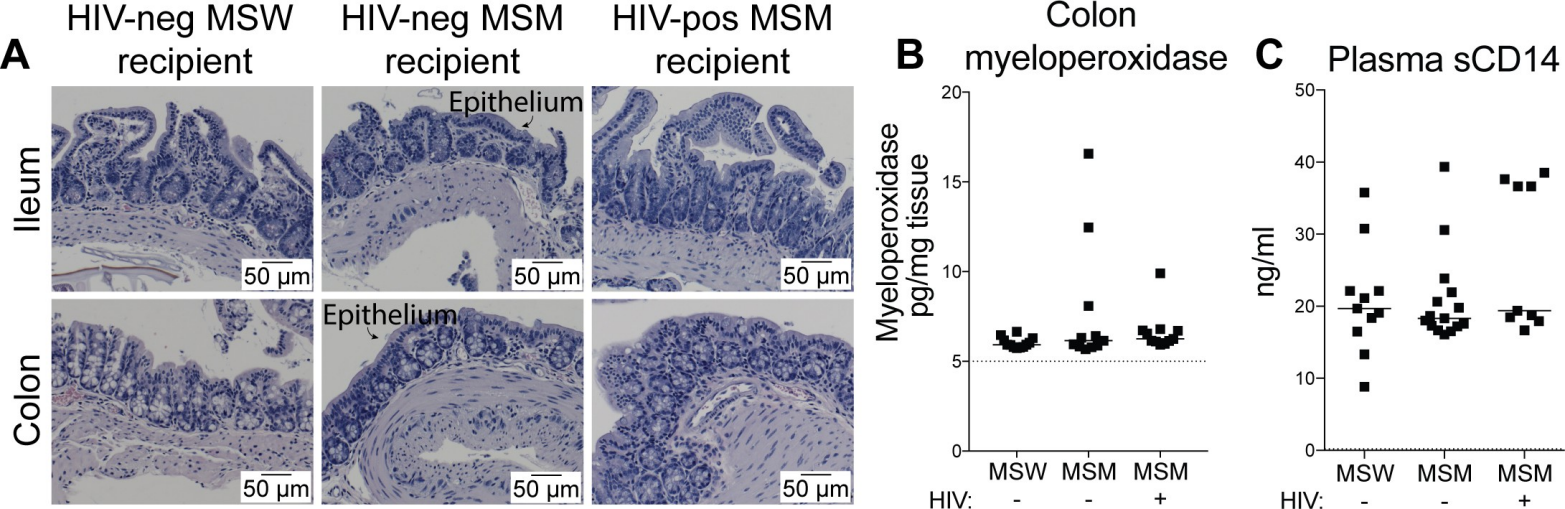
Absence of inflammation, epithelial damage, and bacterial translocation in mouse recipients. (A) Formalin-fixed ileum and colon sections from mouse recipients (5 HIV-negative MSW, 7 HIV-negative MSM, 6 HIV-positive MSM) were paraffin-embedded and H&E stained, and then analyzed by a blinded pathologist for inflammation and epithelial integrity. Representative images of ileum and colon tissue are shown. Arrows point to the epithelium. All tissues analyzed were scored with the minimal inflammation rating of 1, on a scale of 1–4. (B) Myeloperoxidase concentrations measured by ELISA in colon tissues (12 HIV-negative MSW, 11 HIV-negative MSM, 11 HIV-positive MSM). (C) sCD14 levels measured by ELISA in plasma (11 HIV-negative MSW, 15 HIV-negative MSM, 9 HIV-positive MSM). For (B) and (C), each data point represents an individual mouse, lines represent medians, dotted lines represent the limit of detection. Statistical analyses were performed using t-tests to compare groups if data from both groups had normal distributions and Mann-Whitney tests to compare groups if data from at least one group had a non-parametric distribution. No statistically significant differences were detected.

Elevated markers of gut mucosal damage have been reported for MSM and correlated with MSM-associated microbiota [3, 6], and are well known to be associated with HIV infection [33]. To determine if bacteria from the fecal transfer directly caused intestinal damage, ileum and colon tissue samples from 5 HIV-negative MSW, 7 HIV-negative MSM, and 6 HIV-positive MSM mouse recipients were analyzed for histopathology. Tissue health and epithelial integrity was determined to be within normal limits for all mice examined (Fig 5A). Each tissue analyzed was graded with an overall inflammation score on a scale of 1–4 [34], and all tissues were assigned the same minimum inflammation rating of 1. In support of this, myeloperoxidase concentrations (measured in 12 HIV-negative MSW, 11 HIV-negative MSM, and 11 HIV-positive MSM recipients) were not different across groups in the colon (Fig 5B). Levels of soluble CD14 (sCD14) in the blood, a marker of monocyte activation in response to LPS [35] and bacterial translocation across the gut barrier [33], was measured in 11 HIV-negative MSW, 15 HIV-negative MSM, and 9 HIV-positive MSM recipients, and was also not found to be significantly different across groups (Fig 5C). Lack of signs of barrier damage suggest that transfer of microbiota from HIV-negative or HIV-positive men were not sufficient to cause gut tissue injury in mice (at least within 3 weeks of colonization), and that barrier breakdown was not necessary for microbiota from MSM to promote gut T cell activation.

Recapitulation of microbial and immune differences in mouse recipients supports a link between microbiome composition and immune activation in MSM. Microbial-immune correlations consistent across donors and recipients would further strengthen this link. We therefore correlated the relative abundance of gut microbes in donors and recipients with their respective immune data (S5 and S6 Tables). Only immune data significantly different with MSM in either donors or recipients were selected for analyses, since only MSM-associated microbiome differences were detectable in this cohort. Frequencies of blood CD103+ CD8+ T cells in donors, though not significantly different with MSM, were additionally selected for analysis due to their relevance to the gut. Correlations were computed with both the full donor group, as well as with HIV-negative donors only, since HIV may independently drive immune activation in HIV-positive MSM and confound microbial correlations. All correlations that were and were not significant in donors and in mice are shown in tables S5 (donors) and S6 (recipients). After comparing significant correlations across these datasets, we identified 8 microbes that correlated with at least one donor and one recipient immune measurement (Table 1), with 3 of these being negative and 5 of these being positive correlates. Out of these 8 microbes, 5 significantly correlated using the full donor cohort, while 3 significantly correlated using only HIV-negative donors. Two microbes, *Bacteroides uniformis* and *Howardella ureilytica*, significantly differed in relative abundance with MSM in both donors and recipients (S2 Table), and also significantly correlated with immune measurements in both donors and recipients. Though few of the shared microbe-immune correlates were statistically significant with FDR correction, consistency of correlations across humans and mice suggest biological significance, and provide further evidence that immune activation in MSM is influenced by the gut microbiome.

**Table 1:** Microbial correlates with donor/recipient T cell activation and gut homing, and *in vitro* HIV infection.

			Recipients				Donors					FBCs
			Ileum CD8s		Ileum CD4s	Colon CD4s	Blood CD8s				Blood CD4s	In vitro infection
Bacteria	BLAST % identity	RDP database ID	%CD69+	%CD103+	%CD103+	%CD69+	%CD38+ HLADR+	(HIV-) %CD38+ HLADR+	%CD103+	(HIV-) %CD103+	(HIV-) %CD103+	%HIVgag+
*Negatively correlated*												
Akkermansia muciniphila	100.0	S000460661				-0.403, p<0.05			-0.428, p<0.01			
Oscillibacter ruminantium	93.3	S002952856		-0.274, p<0.05			-0.359, p<0.05					
Bacteroides uniformis	100.0	S000001704			-0.415, p<0.0005*						-0.369, p<0.05	
Pseudoflavonifractor capillosus	92.5	S000397238		-0.262, p<0.05		-0.476, p<0.01						-0.385, p<0.05
											** **	** **
*Positively correlated*	* *	* *										
Catenibacterium mitsuokai	97.9	S000022658	0.350, p<0.005	0.296, p<0.05	0.332, p<0.01			0.400, p<0.05	0.432, p<0.01	0.438, p<0.05		
Oscillibacter valericigenes	92.6	S000901479				0.409, p<0.05	0.335, p<0.05	0.417, p<0.05				
Howardella ureilytica	100.0	S000926211				0.404, p<0.05			0.392, p<0.05	0.434, p<0.05		0.361, p<0.05
Desulfovibrio piger	98.6	S000001126			0.251, p<0.05					0.443, p<0.05	0.388, p<0.05	0.375, p<0.05
Clostridium leptum	100.0	S000004442				0.420, p<0.05		0.379, p<0.05				
Holdemanella biformis (1)	98.0	S004459052	0.287, p<0.05	0.290, p<0.05	0.307, p<0.05							0.394, p<0.05
Holdemanella biformis (2)	99.3	S004459052					0.444, p<0.005	0.436, p<0.05	0.607, p<0.0001	0.605, p<0.001	0.461, p<0.05	0.391, p<0.05
Butyricimonas faecihominis	100.0	S004078573							0.328, p<0.05			0.399, p<0.05

Test statistic for Spearman correlations are shown, followed by the p-value, blank spaces indicate non-significant correlations (p>0.05)

* = FDR p-value<0.1

### Microbiota from HIV-negative and HIV-positive MSM stimulate *in vitro* HIV infection

**Fig 6:**
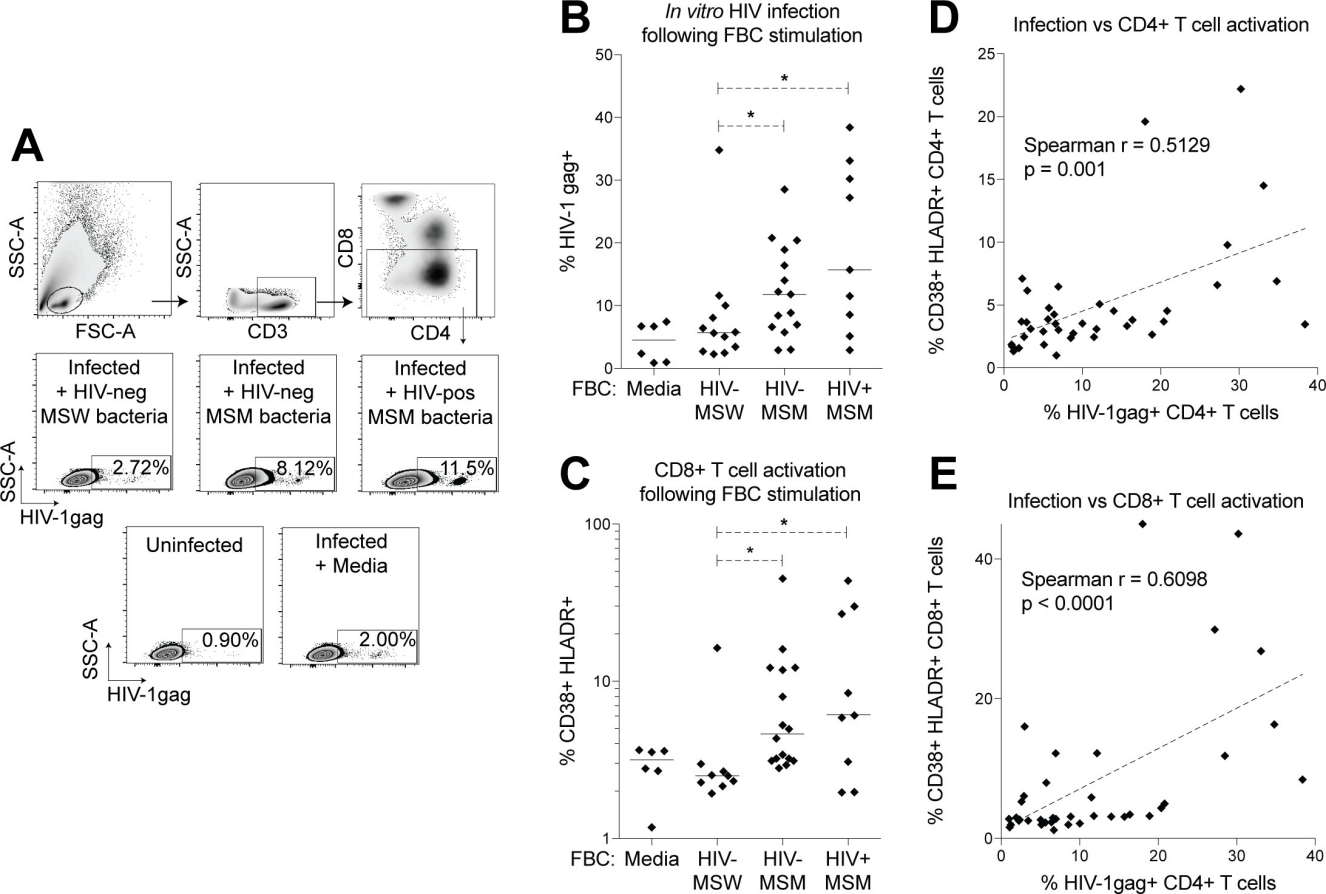
Fecal bacterial communities isolated from HIV-negative and HIV-positive MSM promote HIV infection of primary human lamina propria cells. Primary lamina propria cells (LPCs) were isolated from human jejunum tissue, stimulated with isolated fecal bacterial communities (FBCs) from HIV-negative MSW (n = 12), HIV-negative MSM (n = 15), and HIV-positive MSM (n = 9) donor feces, and infected with HIV_bal_. LPCs were stained for intracellular HIV-1gag expression and T cell activation markers at 5 days post-infection using flow cytometry. (A) Gating strategy for HIVgag+ T cells. (B–C) Comparison of levels of (B) HIV-1gag+ cells and (C) CD38+ HLADR+ CD8+ T cells following infection. Each data point represents the median value of one FBC tested across 3 different individuals’ LPCs. Lines represent medians within groups. Statistical analyses were performed using t-tests to compare groups if data from both groups had normal distributions and Mann-Whitney tests to compare groups if data from at least one group had a non-parametric distribution. * = p<0.05. (D–E) Spearman correlations between frequencies of HIV-1gag+ cells and frequencies of (D) CD38+ HLADR+ CD4+ T cells, and (E) CD38+ HLADR+ CD8+ T cells. Dotted lines represent linear regression analyses.

T cell activation has been correlated with HIV viral load [13], and associated with HIV transmission in women [36]. We therefore investigated whether microbiota from MSM could promote HIV infection *in vitro*. Fecal bacterial communities (FBCs) were isolated from stools of 12 HIV-negative MSW, 15 HIV-negative MSM, and 9 HIV-positive MSM by separating whole intact bacteria in stool from debris using Histodenz columns as previously described [8]. All donors of stool used for FBC isolation are represented in the PBMC immune phenotyping data (Fig 1), while FBCs from 7 HIV-negative MSW, 15 HIV-negative MSM, and 5 HIV-positive MSM were also used as donors in the fecal transplant experiments. HIV-positive individuals used for FBC isolation had a median viral load of 47650 copies/ml and a median CD4 T cell count of 577 cells/μl (S1 Table). Primary human lamina propria cells (LPCs) were stimulated by isolated FBCs, infected with HIV_bal_, and intracellular HIVgag antigen expression was measured after five days. We found that LPCs stimulated with FBCs from HIV-negative and HIV-positive MSM had higher frequencies of HIV-infected cells compared with LPCs stimulated with microbiota from HIV-negative MSW (Fig 6A and 6B). LPCs stimulated with FBCs from HIV-negative and HIV-positive MSM displayed significantly elevated CD8+ T cell activation at the end of infection (Fig 6C), and frequencies of HIV-infected cells significantly correlated with both CD4+ and CD8+ T cell activation of stimulated LPCs (Fig 6D and 6E). FBCs from 7 HIV-negative MSW, 11 HIV-negative MSM, and 8 HIV-positive MSM used in this infection assay were also previously used to stimulate PBMC *in vitro* [8], and frequencies of HIV-infected cells induced by these FBCs significantly correlated with activation levels of peripheral blood CD4+ and CD8+ T cells stimulated by these same FBCs found previously (S8B and S8C Fig). 16S rRNA gene sequencing of FBCs showed that the compositional distinction between MSW and MSM was maintained following bacterial isolation (S9A and S9C Fig). Unweighted UniFrac distances between donor samples and their isolated FBCs were not significantly different across groups (S9B Fig), indicating the average magnitude of stool-FBC compositional shifts were consistent between groups. Specific bacteria that differed with MSM and with HIV in FBCs, and bacteria that significantly changed from stool to FBCs, were previously described [8], and are not presented here. Infection and T cell activation levels trended higher for cells stimulated with HIV-positive MSM FBCs than cells stimulated with HIV-negative MSM FBCs, though these differences were not statistically significant. Finally, six microbes that significantly correlated with *in vitro* HIV infection also correlated with fecal transplant donor and/or recipient immune measurements (Table 1). Interestingly, two sequence variants of *H*. *biformis* correlated with *in vitro* infection, with one significantly correlating with donor blood T cell activation and the other significantly correlating with recipient gut T cell activation. Significant and non-significant correlations between all 16S sequence variants in FBCs with *in vitro* HIV infection are shown in S7 Table. Consistencies were therefore observed between the *in vivo* transplant system and the *in vitro* assays. These data support the findings from the mouse model, and suggest that the gut microbiome in HIV-negative and HIV-positive MSM could impact HIV infection.

## Discussion

Immune differences previously associated with MSM were reproduced in our cohort of fecal donors. We showed elevated blood frequencies of activated CD38+ HLADR+ CD8+ T cells in HIV-negative MSM compared with HIV-negative MSW as previously described [5], as well as increased frequencies of CD103+ CD4+ T cells in HIV-negative MSM, which to our knowledge, is a novel finding. Frequencies of CD103+ CD4+ T cells also correlated with frequencies of CCR5+ CD4+ T cells, suggesting an association between gut homing and CCR5 expression in MSM. Other previously reported immune phenotypes in MSM, such as increased frequencies of gut mucosal Th17 cells and IFN-γ+ TNF-α+ CD8+ T cells [3], increased endotoxemia, and lower blood CD4/CD8 ratios [6] were either not observed or not measured in our cohort. A limitation of our study was the unavailability of immune data from gut tissues in our donors, which would have indeed strengthened associations between our donors and mice. However, our observations in mice are well supported by previous findings of gut T cell activation in MSM [3]. Overall, immune differences associated with MSM in our study were subtle and observed in a small sample size, though the magnitude of these differences reflects what others have found. The subtlety of these differences is also unsurprising, since our cohort of high-risk MSM were healthy individuals without disease. Taken together, the blood T cell profiling presented here has reproduced and expanded on previous knowledge of immune phenotypes associated with MSM, and further emphasize the importance of controlling for MSM in immunological analyses of HIV-positive populations.

Microbiome differences previously associated with MSM were also replicated in our donor cohort (Fig 2C), including increases in *Prevotellaceae*, decreases in *Bacteroidaceae* [2–4], and changes in specific bacterial species such as *Holdemanella biformis* and *Bacteroides uniformis* (S3 Table) [4, 8, 37]. However, previously reported microbiome differences between HIV-negative and HIV-positive MSM, such as increased abundance of *Turicibacter sanguinis* with HIV [4, 8], were not observed in our cohort. Given these results, it was unsurprising that recipient mice reproduced microbiome differences associated with MSM, but showed no significant microbiome differences between recipients of HIV-negative and HIV-positive MSM. Immune activation differences in mouse recipients largely reflected the microbiome composition, with an apparent significant increase in T cell activation associated with recipients of MSM. Immunological differences between HIV-negative and HIV-positive MSM recipients were less clear. Though CD4+ T cell activation in the colon trended higher for recipients of feces from HIV-positive MSM compared with HIV-negative MSM (Fig 3E), this did not reach statistical significance. These trends reflect our previously published results showing FBCs from HIV-positive MSM could promote higher *in vitro* T cell activation than FBCs from HIV-negative MSM [8], and suggest that a larger sample size of donors is needed to investigate immunological differences between mouse recipients of feces from HIV-negative and HIV-positive MSM. However, a small sample size may only partially explain why a significant effect of HIV-associated microbiota was not observed in this study, either in mice or in the *in vitro* infection experiments. In mice, it is possible that HIV-associated microbiota of immunological importance may not have colonized. Furthermore, loss of epithelial integrity is a hallmark of HIV infection and was not evident in these mice. Modeling barrier breakdown in mouse recipients may therefore reveal effects of HIV-associated microbiome differences in the context of HIV-induced disease. In the *in vitro* infection experiments, one finding from our previous work was that differences between HIV-negative MSW and HIV-negative MSM in *in vitro* activation of CD4+ and CD8+ T cells were detected when stimulations were performed with non-autoclaved FBCs but not autoclaved FBCs, whereas the differences in HIV-positive versus HIV-negative MSM were strongly evident with the autoclaved FBCs. Autoclaving was done to ensure that bacteria were killed and that no sporadic growth of antibiotic resistant, aerotolerant bacteria occurred. However, autoclaving could have potentially denatured immune-modulatory compounds. The *in vitro* infection data from this study used non-autoclaved FBCs. Taken together these results suggest that there are differences in the molecular mechanisms of immune activation in HIV-positive and HIV-negative MSM, with those in HIV-positive MSM being more driven by molecular factors that are heat-tolerant.

T cell activation in stool donors and recipients were detected with different markers (CD38 and HLADR in donors, and CD69 and CD103 in recipients) in different compartments (blood and gut), but these markers may have identified related cell populations commonly influenced by gut microbes. In support of this, gut T cell activation in mice significantly correlated with blood T cell activation in donors, and the same bacteria consistently correlated with both donor and mouse immune measurements. Several of these microbial correlates have been previously associated with disease and/or known to have immune-modulating potential. This includes *Holdemanella biformis* (formerly known as *Eubacterium biforme*), which is increased with MSM in donors and recipients, and has been previously associated with stimulation of inflammatory cytokine production and correlated with T cell activation *in vitro* [8, 38]. *Bacteroides uniformis*, a Treg and IL10 inducer [39] that has been found to be reduced in patients with Crohn’s disease [40], is also reduced with MSM in our donors and recipients. *Akkermansia muciniphila*, a mucolytic bacteria associated with health and disease depending on context, has been found to be reduced in patients with ulcerative colitis and Crohn’s disease [41], and was negatively correlated with CD4+ T cell activation in recipient colons and mucosal homing CD8+ T cells in donor blood. Finally, *Desulfovibrio piger*, a sulfate-reducing bacteria associated with inflammatory bowel disease [42], was positively correlated with gut homing T cells in recipients and donors. Thus, bacteria correlated with immune function in our dataset is consistent with previous findings. Organisms identified in these correlations are ideal candidates for further investigation of their immune-modulating properties in HIV-negative and HIV-positive MSM.

Transfer of the microbiome from human donors to mice was imperfect, as significant differences between MSW and MSM were not reproduced in the mice. Chief among these was *Prevotella*, which was lost from recipients of MSM but not recipients of MSW. Though two of the *P*. *copri* sequence variants identified may be unique features of *Prevotella*-rich MSW that are better adapted for mice, the inability of the third variant to colonize mice in the context of an MSM microbiome suggests that the broader community structure, which is different between MSM and *Prevotella*-rich non-MSM [4], influences *Prevotella* colonization. Since *Prevotella* has been associated with inflammation in other studies, including being positively correlated with gut immune activation in HIV-positive individuals [43], and being associated with rheumatoid arthritis [44], successful colonization in recipients of MSM stool may have driven an even larger effect of immune activation. Alternatively, *Prevotella* may not be a major influencer of immune activation, since it did not induce noticeably higher activation in either monocolonized mice or *Prevotella*-rich MSW fecal recipients. Additionally, *Prevotella* variants were also not identified as significant correlates with immune measurements in donor blood. Another intriguing hypothesis is that *Prevotella* may only promote inflammation in a diseased state. Indeed, in mice with DSS-induced colitis, the presence of *P*. *copri* (identical to *P*. *copri* variant 3 in this study) led to more severe inflammation [45]. Inducing colitis to mimic gut disease during HIV infection in mouse recipients of *Prevotella*-rich MSW may reveal similar effects. Importantly, these results demonstrate that despite prominent microbiome differences like *Prevotella* abundance not being reproduced in mouse recipients, compositional distinction between MSW and MSM was maintained in mice by successful transfer of critical immune-modulating bacteria.

The ability of microbiota from HIV-negative and HIV-positive MSM to promote HIV infection *in vitro* suggests that the gut microbiome composition could contribute to HIV infection and disease progression in MSM. There is evidence that the vaginal microbiome is a risk factor for HIV transmission in women: vaginal bacteria modulate immune activation in vaginal tissue [9], bacterial vaginosis has been linked to vaginal HIV transmission [11, 12], and specific microbes in the vaginal microbiome have been associated with inflammation and increased HIV acquisition [36]. Thus, it is possible that the gut microbiome may also contribute to risk in MSM by promoting immune activation in gut tissues. Increased frequencies of CD69+ and CD103+ T cells in mouse recipients of MSM suggest there are more tissue-resident memory CD4+ T cells in the gut [31], and memory CD4+ T cells are known to be the primary targets of HIV infection [46]. Though these cells were assessed in the ileum and colon rather than the rectum, it is clear they are driven by the gut microbiome–which was linked to both rectal T cell activation and rectal SHIV transmission in macaques [47]. Furthermore, gut microbes that correlated with T cell measures in the ileum and colon of mouse recipients also correlated with *in vitro* HIV infection. Therefore, microbes that influence T cell activation along the length of the gut likely also impact HIV infection at the specific site of transmission. These findings are strong rationale for conducting longitudinal studies examining the association between gut microbiome composition and HIV transmission in populations of high-risk MSM.

In conclusion, this study provides evidence of a direct link between the gut microbiome and immune activation observed in high-risk MSM, by demonstrating that both microbiome and immune phenotypes in MSM donors are transferrable to mouse recipients through fecal transplantation. Mice were receptive of colonization by key immune-influencing microbes that induced immunological differences associated with MSM donors, suggesting the gut microbiome influences immune activation in MSM.

## Materials and methods

### Study subjects

Stool samples were obtained from HIV-negative MSW, high-risk HIV-negative MSM, and HIV-positive MSM not on antiretroviral therapy. Male HIV-positive individuals enrolled in the study were determined to be MSM using a behavioral questionnaire. High-risk MSM were recruited from a high-risk cohort assembled for a study of a candidate HIV-1 preventative vaccine [48]. Designation of high-risk was according to a number of different behaviors including 1) a history of unprotected anal intercourse with one or more male or male-to-female transgender partners 2) anal intercourse with two or more male or male-to-female transgender partners and 3) being in a sexual relationship with a person who has been diagnosed with HIV. All enrolled individuals live in metropolitan Denver. Other inclusion criteria used were: 18–70 years old, body mass index (BMI) between 21–29 mg/kg^2^ and weight stable for at least 3 months; for HIV-positive individuals, <10 days of ART treatment at any time, or previously on ART but off treatment for the previous 6 months, prior to stool and blood collection; liver and kidney function tests within normal range. Exclusion criteria used were: weight <110 pounds; received antibiotics within the prior 90 days; active gastrointestinal disease; history of bowel resection. All subjects used in this study were previously characterized for diet and sexual behavior as part of a larger cohort; diet or any particular sexual behavior that we measured were not identified as driving factors of the most prominent microbiome differences between MSM and MSW [4].

### Ethics statement

Written informed consent was obtained from healthy HIV-negative and HIV-positive individuals for use of stool. The study protocol was approved by the Colorado Multiple Institution Review Board (COMIRB No: 14–1595). All subjects were adults. Mice were handled in accordance with the recommendations in the National Institutes of Health Guide for the Care and Use of Laboratory Animals and protocols were approved by the University of Colorado Institutional Animal Care and Use Committee Permit Number 00097. Primary human lamina propria cells were collected from otherwise discarded tissues from gut resection surgery, and was determined to not be human subject research under protocol number 14–1184 approved by the University of Colorado Multiple Institutional Review Board. Tissues were obtained from anonymous adult patients who were not asked for consent.

### PBMC immune phenotyping

PBMC were isolated from patient blood by Ficoll-Paque (GE), and cryopreserved and stored in liquid nitrogen before analysis. PBMC were thawed, and 5 x 10^5^ cells were stained for 30 min at 4° C with the following antibodies: CD3-APC-Cy7 (OKT3, Biolegend), CD4-PerCP-Cy5.5 (OKT4, Biolegend), CD8-APC (SK1, Biolegend), CD38-BV421 (HIT2, Biolegend), CD69-BV510 (FN50, Biolegend), HLADR-FITC (L243, Biolegend), CD103-PE (BerACT8, Biolegend), CCR5-PE-Cy7 (J418F2, Biolegend). Cells were then washed in FACS buffer and fixed in 1% paraformaldehyde before being acquired on a BD FACS Canto II. 1 x 10^6^ PBMC were stimulated with a PMA/ionomycin cell stimulation cocktail (eBioscience), and treated with a protein transport inhibitor cocktail (eBioscience) for 4 h at 37° C, 5% CO_2_. Cells were then stained for 30 min at 4° C with the following antibodies: CD3-APC-Cy7 (OKT3, Biolegend), CD4-PerCP-Cy5.5 (OKT4, Biolegend), CD8-APC (SK1, Biolegend). Cells were washed and treated with fix/perm buffer (eBioscience) for 30 min at 4° C, before being stained for intracellular cytokines for 30 min at 4° C with the following antibodies: IFN-γ-PE (4SB3, Biolegend), TNFα-APC-Cy7 (MAb11, Biolegend). Cells were then washed in FACS buffer and fixed in 1% paraformaldehyde before being acquired on a BD FACS Canto II.

### Gavages

1.5 grams of frozen feces stored at -80° C for up to one year from each donor was thawed in an anaerobic chamber and homogenized in 3 ml of anaerobic PBS, using a syringe handle. Fecal solutions were then filtered through a 100 μm nylon filter into a 50 ml conical tube. Tubes were sealed before removing from the anaerobic chamber and transferred to the mouse facility within 1 hour of thawing. 200 μl of each fecal solution was used to gavage each mouse.

### Bacterial load in donor stool

2 mg of stool from donors were homogenized in PBS, and bacteria were then isolated using two sequential rounds of sucrose density centrifugation. Isolated bacterial cells were stained with thiazol orange and propidium iodide, and acquired by a FACS Canto II (BD) flow cytometer in the presence of counting beads. Data were processed using Flowjo software, and the concentration of live bacterial cells per mg of stool was calculated.

### Gnotobiotic mice

Germ-free C57/BL6 mice were purchased from Taconic and bred and maintained in flexible film isolator bubbles. Male mice between 6–8 weeks of age were gavaged with fecal solutions prepared from donor fecal samples and housed individually (to ensure mice would not contaminate each other) following gavage for 3 weeks in a Tecniplast iso-positive caging system, with each cage having HEPA filters and positive pressurization for bioexclusion. Feces were collected from mice at day 0 for 16S rRNA gene sequencing to confirm germ-free status. 16S sequencing of feces from day 7 and day 14 showed relatively little variation in composition from day 21 (S2 Fig). Mice were euthanized at 21 days post gavage using isoflurane overdose and all efforts were made to minimize suffering. Blood from euthanized animals was collected using cardiac puncture and cells were pelleted in K2-EDTA tubes; plasma was then aliquoted and stored at -80° C. Data was combined from 10 batches of mice analyzed independently, with at least 4 mice per batch, and at least two donor groups represented in each batch.

### Mouse ileum and colon single cell preparation

Ileum and colon tissues from mice at 21 days post gavage were collected for immune phenotyping and histology. Tissues were dissected length-wise and feces were washed away using PBS. 1–5 mm slices of ileum and colon closest to the cecum were taken from a subset of mice and fixed in 10% formalin and reserved for histology or ELISA. The remaining tissue was then washed in a PBS solution containing 1% EDTA for two 5 min intervals on a vortexer. Tissues were washed with PBS and strained through a 100 μm nylon filter in between intervals. Tissues were then washed for a third time in plain 1X PBS. Tissues were then minced using an octoMACs tissue dissociator (Miltenyi) and digested in 2.5 ml of a solution of complete RPMI (10% FBS, 1% PSG) containing 1 mg/ml collagenase D type I (Worthington Biotech) at 37° C, 5% CO_2_. Released cells were quenched with cold complete media, filtered through a 70 μm nylon filter and washed with complete media before staining for flow cytometry.

### Mouse immune phenotyping

0.5–1 x 10^6^ ileum and colon cells from each mouse were stained in FACS buffer for 30 min at 4° C with the following antibodies: CD3-PerCP-Cy5.5 (17A2, Biolegend), CD4-FITC (RM4-4, eBioscience), CD8-BV510 (53–6.7, Biolegend), CD69-PE-Cy7 (h1.2F3, eBioscience), CD103-PE-Dazzle-594 (2E7, Biolegend). Mesenteric lymph node cells were additionally stained with CD44-AlexaFluor-700 (IM7, eBioscience), and CD62L-APC-eFluor780 (MEL-14, eBioscience). Cells were then washed with FACS buffer and fixed in 1% paraformaldehyde before being acquired on a BD LSRii flow cytometer. Colon samples that had less than 1,000 T cells were excluded. Plasma samples were thawed and diluted 1:10 before being assayed for sCD14 by ELISA (R&D Systems) according to the manufacturer’s instructions. Ileum and colon tissues cultured for 24 h at 37° C, 5% CO_2_ in complete RPMI were digested with CellLytic MT solution with proteinase inhibitor (SIGMA), and assayed for myeloperoxidase concentrations by ELISA (R&D Systems). ELISA plates were read using a Vmax Kinetic plate reader (Molecular Devices). Tissue sections from the distal end of the ileum and proximal end of the colon were processed for histology and H&E stained. Scoring [34] was performed by a pathologist blinded to experimental conditions.

### Stool collection and FBC preparation

Stool samples were collected by the patient, both on a sterile swab and with a sterile scoop within 48 hours of a clinic visit. Samples were stored immediately in a cooler with -20°C freezer packs. After delivery to the clinic, the swab was subsequently stored at -80° C to await DNA extraction. Isolation of fecal bacterial communites (FBCs) from stool was described previously [8]. Briefly, two grams of feces were homogenized in sterile PBS and filtered through a 100uM filter. Homogenate was then subjected to two density gradient centrifugations with 80% Histodenz (Sigma). Bacterial layers were collected after each spin, visualized with a light microscope, and quantified by flow cytometry using a bead counting kit (BD Biosciences). FBCs were stored at -80° C before use in *in vitro* assays.

### 16S rRNA gene sequencing

16S rRNA targeted sequencing was conducted according to earth microbiome project standard protocols (http://www.earthmicrobiome.org). DNA was extracted from donor stool swabs, mouse fecal pellets, and from a 250 μL aliquot of FBC isolates using the standard PowerSoil protocol (QIAGEN). PCR amplification of the extracted DNA, along with water controls, was conducted with barcoded primers targeting the V4 region of the 16S rDNA gene (515F-806R; FWD:GTGCCAGCMGCCGCGGTAA; REV:GGACTACHVGGGTWTCTAAT). Amplified DNA was quantified using a PicoGreen assay (Invitrogen) so equal amounts of DNA from each sample could be pooled and cleaned using the UltraClean PCR Clean-up protocol (Qiagen). The final DNA pool was sequenced using the Illumina MiSeq platform (San Diego, CA) using the V2 2x250 kit.

### Sequencing analysis

Raw sequences were assigned to samples based on their barcodes using QIIME 2.6 [49]. The libraries were denoised and grouped by sequence variants using dada2 1.2.2. Samples contained from 21,357 to 159,296 sequences; analyses that contained donor and mouse recipient feces were conducted on the standardized sequence number of 21,357, analyses that contained FBC isolates and their original stool were conducted on the standardized sequence number of 21,812, and analyses that contained mice monocolonized with *P*. *copri* and *B*. *uniformis* were conducted on the standardized sequence number of 31,812 –these numbers were determined as the minimum read number acquired for a single sample in each independent analysis. Sequence variants were classified taxonomically using the RDP classifier [50] trained on the greengenes 3_8 taxonomic database [51]. Principal Coordinates Analysis of unweighted UniFrac distance matrices, and rendering of taxa bar charts, were conducted using QIIME 2.6. For immune correlations, comparisons of relative abundance between groups, and *P*. *copri* variant analysis, sequence variants not observed in at least 20% of the samples in each analysis were removed.

### LPC collection

Macroscopically healthy jejunum tissues from patients undergoing elective gut resection surgery were digested to release lamina propria cells (LPCs). Briefly, tissues were trimmed of fat and the muscularis layer was removed using scissors. Trimmed tissues were incubated on a rocker with a 1.6 mM dithiolthreitol (DTT) PBS solution for 45 min at 37° C, and then with a 1 mM EDTA PBS solution for 60 min at 37° C. Tissues were then washed with PBS on a vortexer, minced using scissors, and incubated for three 45 min intervals with a digestion solution of complete RPMI (10% HS, 1% PSG) containing 1 mg/ml collagenase D (Sigma-Aldrich) and 10 μg/ml DNase I (Sigma-Aldrich). Released cells were collected and filtered through a 70 μm nylon filter after each digestion interval. Cells from each interval were pooled together, RBC lysed, and cryopreserved.

### *In vitro* LPC infection

LPCs were thawed in complete RPMI containing 10 μg/ml DNase I (Sigma-Aldrich), and washed again with complete RPMI before plating. 5 x 10^5^ LPCs were plated per well in a 96-well round bottom plate. Bacterial cells were added to specified wells at 5:1 bacteria:LPC ratio. HIV_bal_ passaged in Molt4 T cells and quantified by qPCR were then added to each well at 10^7^ HIV RNA copies/well. Cells were incubated at 37° C for 24 h to allow for infection, and then washed twice with complete RPMI to remove cell-free virus. Bacteria were added back to each well at a 5:1 ratio after washing, and cells were incubated for another 4 days at 37° C. LPCs were then stained with anti-CD3-APC-Cy7, CD4-PerCP-Cy5.5, CD8-APC, HLADR-FITC, CD38-BV421, CD69-BV510, and CCR5-PE-Cy7. Cells were then washed and treated with fix/perm buffer (eBioscience) for 30 min at 4° C, and stained with anti-HIVgag-PE (KC57, Fisher) for 1 h at 4° C. Cells were then fixed in 1% paraformaldehyde and acquired using a FACS Canto II flow cytometer (BD Biosciences). Unstimulated, uninfected cells were used as controls to gate on HIVgag+ cells.

### Statistics

Immune data were compared between groups using t-tests if data from both groups passed the D’Agostino and Pearson normality test, and Mann-Whitney tests if data from at least one group did not pass the normality test. All immune data were analyzed using Prism 7 (Graphpad). Comparisons of bacterial relative abundances between groups were performed using non-parametric t-tests on non-transformed data. Spearman correlations were used for all correlation analyses. Replicate recipient mice were treated as independent observations for all microbiome comparisons and correlations. Corrected p-values were determined with the False Discovery Rate (FDR) technique of Benjamini and Hochberg. All statistical analyses involving 16S sequencing data were performed using QIIME 1.9.

## Supporting information

S1 Fig Blood T cell phenotypes in HIV-negative MSW, HIV-negative MSM, and HIV-positive MSM. PBMC from HIV-negative MSW (n = 18), HIV-negative MSM (n = 19), and HIV-positive MSM (n = 13) were stained for T cell markers and intracellular cytokines and analyzed by flow cytometry. (A-B) Comparison of frequencies of CD69+ (A) CD8+ and (B) CD4+ T cells. (C-D) PBMC stimulated with PMA/ionomycin were stained for intracellular cytokines. Frequencies of TNF-α+ IFN-γ+ (C) CD8+ and (D) CD4+ T cells. (E) Frequencies of CCR5+ CD4+ T cells. (F) %CD4+/%CD8+ T cell ratio. Each data point represents an individual, and lines represent median values. Statistical analyses were performed using t-tests to compare groups if data from both groups had normal distributions and Mann-Whitney tests to compare groups if data from at least one group had a non-parametric distribution. **** = p <0.0001, *** = p<0.001, ** = p<0.01, * = p<0.05.
(TIF)

S2 Fig Longitudinal analysis of microbiome composition in mouse recipients. Compositions of mouse recipients at 7, 14, and 21 days post-gavage were assessed by 16S rRNA gene sequencing, and representative data from 5 HIV-negative MSW recipients are shown. Unweighted Unifrac PCoA plots are colored by each unique mouse recipient (A) and by timepoint (B).
(TIF)

S3 Fig Relative abundance of bacterial families in donors and recipients. (A) Taxa bar chart showing relative abundance of bacterial families in each donor and their respective recipient(s), side-by-side. Red boxes along the top of the chart represent donors, with subsequent black boxes representing their mouse recipient(s)–multiple black boxes represent replicate recipients. (B) Taxa bar chart showing the mean relative abundance of bacterial families within each donor and recipient group. Legends identify the top 20 most abundant families across all samples. f__ = unclassified bacterial family.
(TIF)

S4 Fig Characterization of Prevotella copri variants in donors and mice. *Prevotella* sequences in mice were comprised of only three *Prevotella copri* variants (1–3), which colonized only two mice. Six other *P*. *copri* variants (4–9) were present in at least 20% of MSM donors but did not colonize mice. (A) Relative abundance of each *P*. *copri* variant in mouse recipients (squares). Red and gray squares were recipients of *Prevotella*-rich MSW. (B) Relative abundance of each *P*. *copri* variant in donors and their recipients. Lines connect donors to their recipients. Spheres represent an individual donor, and squares represent a mouse colonized with an individual stool; a representative mouse is shown for stools tested in replicate. Blue circles represent MSM (both HIV-negative and HIV-positive) donors, while black circles represent HIV-negative MSW donors.
(TIF)

S5 Fig Colonization fidelity in mouse recipients. (A) Unweighted Unifrac was used to analyze relative distance in microbiome composition between donors and recipients. Each data point represents the relative vector distance between a donor/recipient pair. (B) 16S sequencing data was filtered to exclude sequence variants not present in at least 20% of all donors and recipients. Following filtering, the percentage of unique sequence variants identified in each donor that were present in their recipient was calculated, and is represented as a single data point. A representative recipient replicate was used when applicable for both (A) and (B). Lines represent median values. Statistical analyses were performed using t-tests. No statistically significant differences were detected.
(TIF)

S6 Fig Colon CD8+ T cell activation in mouse recipients. Colon tissues were collected from mouse recipients at 21 days post gavage and analyzed for frequencies of CD69+ (left) and CD103+ (right) CD8+ T cells. Lines represent median values and each point is the mouse recipient of a unique stool donor. Statistical analyses were performed using Mann-Whitney tests.
(TIF)

S7 Fig T cell activation in mice colonized by Prevotella in pure culture or in a mixed population. (A) Mice were monocolonized with either *Bacteroides uniformis* or *Prevotella copri*, and frequencies of CD4+ T_em_ cells in mesenteric lymph nodes were assessed at 21 days post-gavage. (B) Immune data from Fig 3 was modified to distinguish MSW stool recipients successfully colonized by Prevotella (red squares). Each data point represents a single mouse recipient of monoculture or stool. Lines represent medians. Statistical analyses were performed using t-tests to compare groups if data from both groups had normal distributions and Mann-Whitney tests to compare groups if data from at least one group had a non-parametric distribution. **** = p <0.0001, *** = p<0.001, ** = p<0.01, * = p<0.05.
(TIF)

S8 Fig Correlations of in vivo and in vitro immune data. (A–B) Immune data from donors were correlated with immune data from mouse recipients. (A) Frequencies of blood CD38+ HLADR+ CD8+ T cells in all donors (plotted in log scale but correlated using linear data) were correlated with frequencies of ileum CD69+ CD8+ T cells in their recipients, and (B) frequencies of blood CD103+ CD4+ T cells in HIV-negative donors were correlated with frequencies of colon CD69+ CD4+ T cells in their recipients. Each data point represents a donor-recipient pair, with representative recipients used for donors tested in replicates. (C–D) *In vitro* infection data were correlated with previously published data [8] of *in vitro* FBC stimulation of primary human PBMC. HIV infection levels in LPCs stimulated by FBCs correlated with CD4+ T cell (A) and CD8+ T cell activation (B) in PBMC stimulated with the same FBCs. Each point represents an individual FBC that was used in both the infection assay in this study, and in previously published *in vitro* PBMC stimulations. Spearman r values and p values are shown.
(TIF)

S9 Fig Characterization of microbiome composition in FBCs. 16S rRNA gene sequencing was used to characterize the bacterial composition of original stool samples used for bacterial isolation, and fecal microbial communities (FBCs) following bacterial isolation. Sequencing data for five FBCs were not of high enough quality to be included in the final analyses. (A) Unweighted UniFrac clustering of stool (large dots) and FBC (small dots) compositions, with a line connecting each FBC to their original stool. Green dots are HIV-negative MSW, red dots are HIV-negative MSM, and blue dots are HIV-positive (ART-naïve) MSM. (B) Unweighted UniFrac distance between each FBC and their original stool were calculated and compared across groups. Each data point represents relative distance between an individual stool-FBC pair. Statistical analyses were performed using t-tests. No statistically significant differences were detected between groups. (C) Taxa bar chart showing the relative abundance of bacterial families in FBCs and original stools. Legend identifies the top 20 most abundant families across all samples. f__ = unclassified bacterial family.
(TIF)

S1 Table Study subject characteristics. For race and ethnicity, values are numbers of white/white-hispanic/black/asian study subjects included in each analysis. For CD4 count and nadir, values are cells/μl. For viral load, values are copies/ml.
(XLSX)

S2 Table Bacteria significantly different with MSM in recipients and donors. 16S sequence variants significantly different in relative abundance with MSM were identified in mouse recipients. 5/6 variants were also significantly different with MSM in donors. ns = not significant.
(XLSX)

S3 Table 16S sequence variants significantly and not significantly different in relative abundance with MSM and with HIV in recipients and donors. Four comparisons are shown, each one in an individual tab: (1) HIV-neg MSW vs HIV-neg MSM in recipients, (2) HIV-neg MSM vs HIV-pos MSM in recipients, (3) HIV-neg MSW vs HIV-neg MSM in donors, and (4) HIV-neg MSM vs HIV-pos MSM in donors. Non-parametric t-tests were performed for all sequence variants. FDR_P = FDR corrected p-values, Bonferroni_P = Bonferroni corrected p-values. Differences with FDR_P<0.1 are highlighted.
(XLSX)

S4 Table Bacterial families different in relative abundance between donors and recipients. The four tabs in this table are data from non-parametric t-tests performed for donors vs recipients for: (1) all groups, (2) HIV-neg MSW, (3) HIV-neg MSM, and (4) HIV-pos MSM. Families that significantly decreased in mouse recipients are highlighted in red and families that significantly increased in mouse recipients are highlighted in green (FDR p-value<0.1). Non-parametric t-tests were performed for all bacterial families identified. FDR_P = FDR corrected p-values, Bonferroni_P = Bonferroni corrected p-values. f__ = unclassified bacterial family.
(XLSX)

S5 Table 16S sequence variants significantly and not significantly correlated with immune measurements in donors. Sequence variants identified in stool of all donors were correlated with their blood immune measurements: (1) %CD38+ %HLADR+ CD8+ T cells, and (2) %CD103+ CD8+ T cells. Sequence variants identified in stool of HIV-negative donors only were correlated with their blood immune measurements: (3) %CD38+ %HLADR+ CD8+ T cells, (4) %CD103+ CD8+ T cells, and (5) %CD103+ CD4+ T cells. Spearman correlations with each immune measurement were performed for all sequence variants. pval_fdr = FDR corrected p-values, pval_bon = Bonferroni corrected p-values. Correlates with pval<0.05 are highlighted.
(XLSX)

S6 Table 16S sequence variants significantly and not significantly correlated with immune measurements in recipients. Sequence variants identified in stool of mouse recipients were correlated with their immune measurements: (1) %CD69+ CD8+ T cells in the ileum, (2) %CD103+ CD8+ T cells in the ileum, (3) %CD103+ CD4+ T cells in the ileum, and (4) %CD69+ CD4+ T cells in the colon. Spearman correlations with each immune measurement were performed for all sequence variants. pval_fdr = FDR corrected p-values, pval_bon = Bonferroni corrected p-values. Correlates with pval<0.05 are highlighted.
(XLSX)

S7 Table 16S sequence variants in FBCs significantly and not significantly correlated with in vitro HIV infection. Sequence variants identified in FBCs were correlated with their stimulated %HIVgag+ cells in the *in vitro* infection assay. Spearman correlations were performed for all sequence variants. pval_fdr = FDR corrected p-values, pval_bon = Bonferroni corrected p-values. Correlates with pval<0.05 are highlighted.
(XLSX)
