# Supplementary figures and images for: Gut microbiota from high-risk men who have sex with men drive immune activation in gnotobiotic mice and in vitro HIV infection
Source: PLoS Pathog. 2019 Apr 4;15(4):e1007611. doi: 10.1371/journal.ppat.1007611 (PMC6448819; doi:10.1371/journal.ppat.1007611)

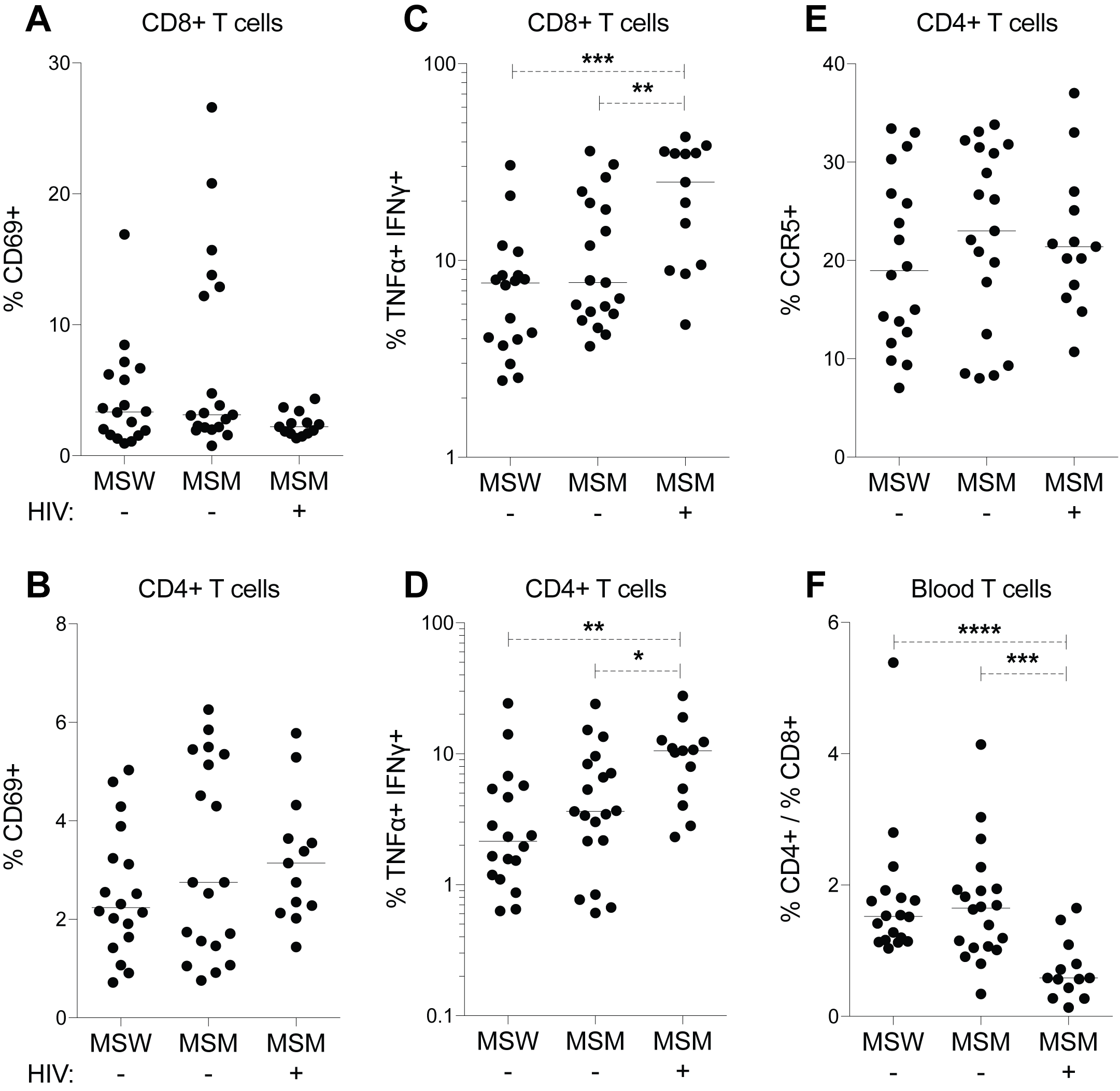

Supplement: S1 Fig — PBMC from HIV-negative MSW (n = 18), HIV-negative MSM (n = 19), and HIV-positive MSM (n = 13) were stained for T cell markers and intracellular cytokines and analyzed by flow cytometry. (A-B) Comparison of frequencies of CD69+ (A) CD8+ and (B) CD4+ T cells. (C-D) PBMC stimulated with PMA/ionomycin were stained for intracellular cytokines. Frequencies of TNF-α+ IFN-γ+ (C) CD8+ and (D) CD4+ T cells. (E) Frequencies of CCR5+ CD4+ T cells. (F) %CD4+/%CD8+ T cell ratio. Each data point represents an individual, and lines represent median values. Statistical analyses were performed using t-tests to compare groups if data from both groups had normal distributions and Mann-Whitney tests to compare groups if data from at least one group had a non-parametric distribution. **** = p <0.0001, *** = p<0.001, ** = p<0.01, * = p<0.05. (TIF) [file ppat.1007611.s001.tif]

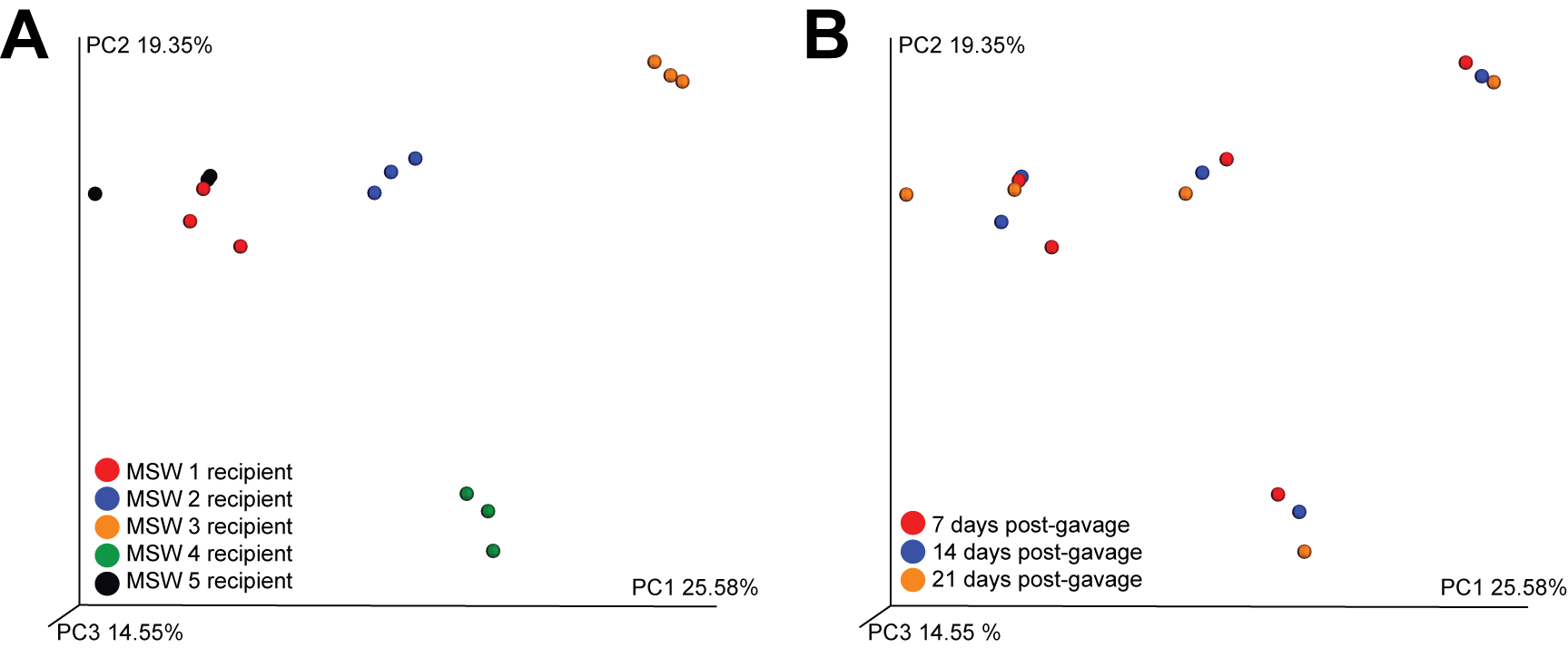

Supplement: S2 Fig — Compositions of mouse recipients at 7, 14, and 21 days post-gavage were assessed by 16S rRNA gene sequencing, and representative data from 5 HIV-negative MSW recipients are shown. Unweighted Unifrac PCoA plots are colored by each unique mouse recipient (A) and by timepoint (B). (TIF) [file ppat.1007611.s002.tif]

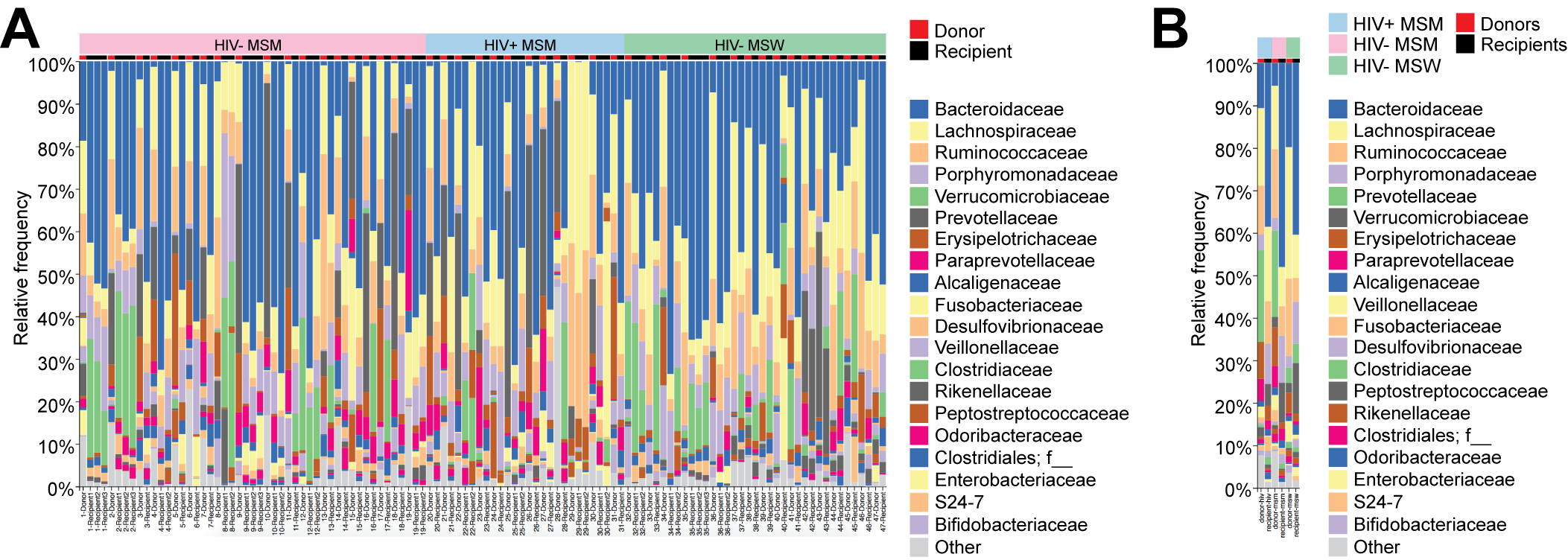

Supplement: S3 Fig — (A) Taxa bar chart showing relative abundance of bacterial families in each donor and their respective recipient(s), side-by-side. Red boxes along the top of the chart represent donors, with subsequent black boxes representing their mouse recipient(s)–multiple black boxes represent replicate recipients. (B) Taxa bar chart showing the mean relative abundance of bacterial families within each donor and recipient group. Legends identify the top 20 most abundant families across all samples. f__ = unclassified bacterial family. (TIF) [file ppat.1007611.s003.tif]

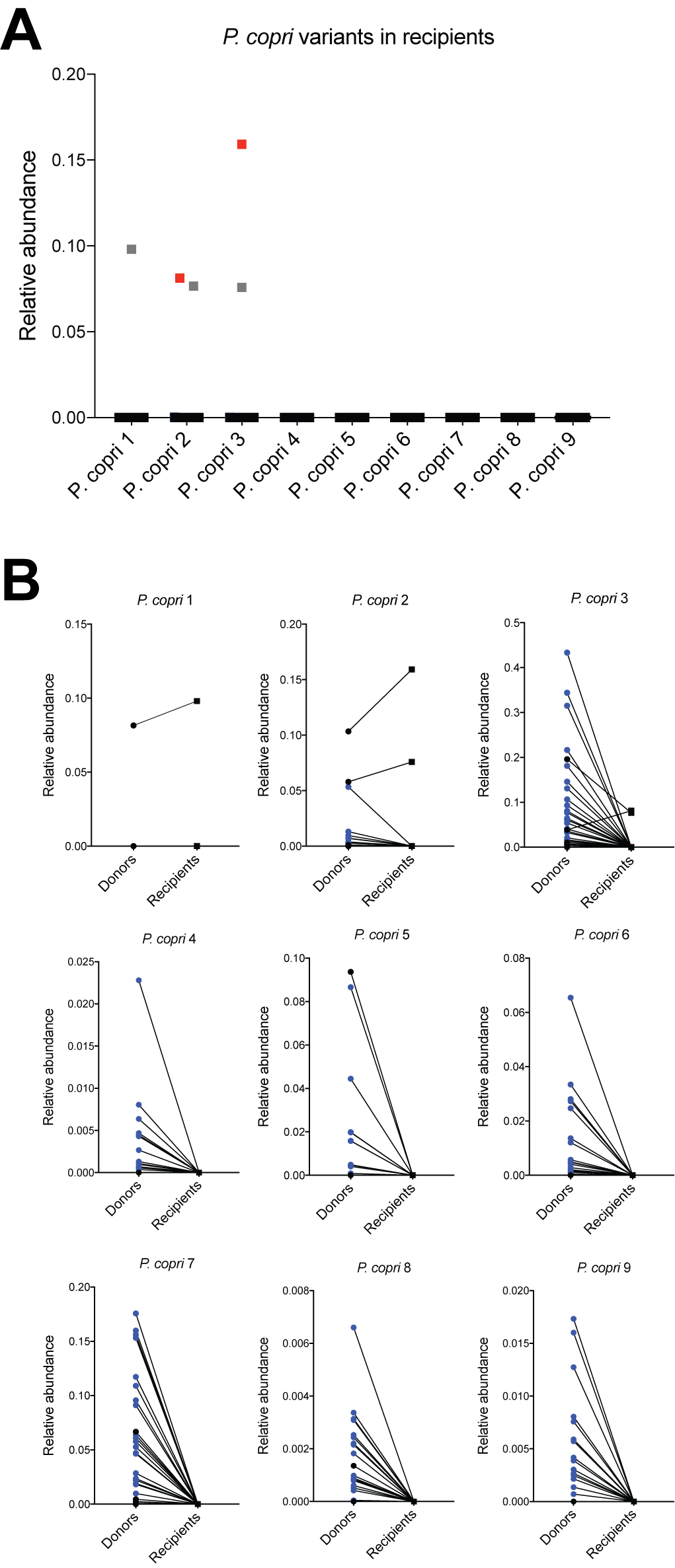

Supplement: S4 Fig — Prevotella sequences in mice were comprised of only three Prevotella copri variants (1–3), which colonized only two mice. Six other P. copri variants (4–9) were present in at least 20% of MSM donors but did not colonize mice. (A) Relative abundance of each P. copri variant in mouse recipients (squares). Red and gray squares were recipients of Prevotella-rich MSW. (B) Relative abundance of each P. copri variant in donors and their recipients. Lines connect donors to their recipients. Spheres represent an individual donor, and squares represent a mouse colonized with an individual stool; a representative mouse is shown for stools tested in replicate. Blue circles represent MSM (both HIV-negative and HIV-positive) donors, while black circles represent HIV-negative MSW donors. (TIF) [file ppat.1007611.s004.tif]

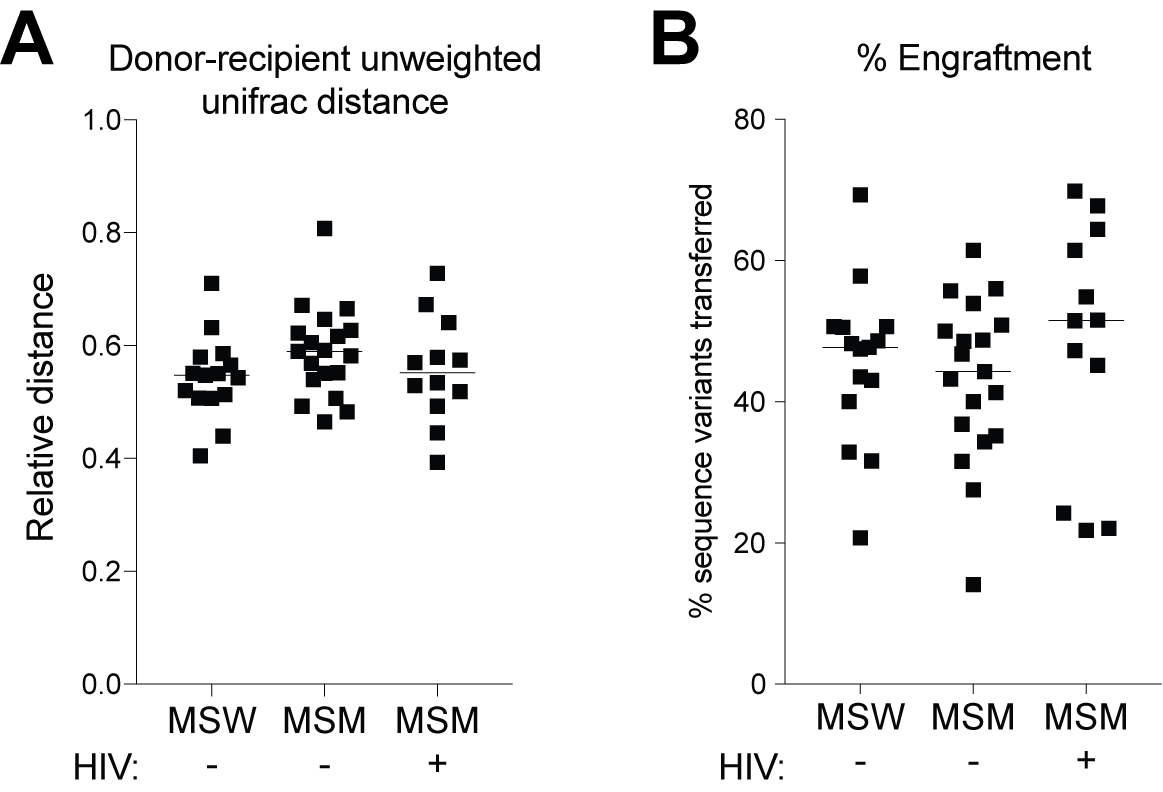

Supplement: S5 Fig — (A) Unweighted Unifrac was used to analyze relative distance in microbiome composition between donors and recipients. Each data point represents the relative vector distance between a donor/recipient pair. (B) 16S sequencing data was filtered to exclude sequence variants not present in at least 20% of all donors and recipients. Following filtering, the percentage of unique sequence variants identified in each donor that were present in their recipient was calculated, and is represented as a single data point. A representative recipient replicate was used when applicable for both (A) and (B). Lines represent median values. Statistical analyses were performed using t-tests. No statistically significant differences were detected. (TIF) [file ppat.1007611.s005.tif]

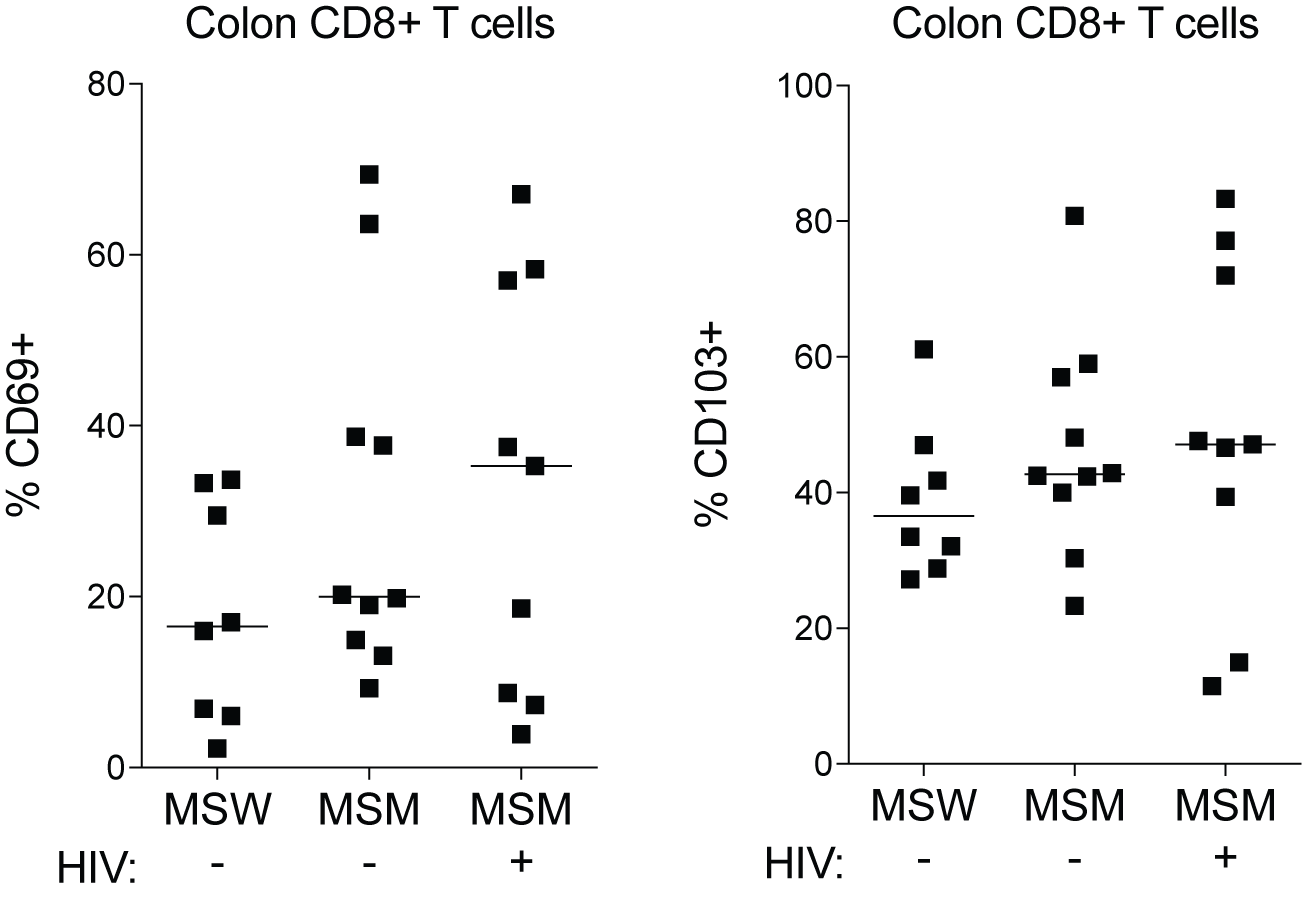

Supplement: S6 Fig — Colon tissues were collected from mouse recipients at 21 days post gavage and analyzed for frequencies of CD69+ (left) and CD103+ (right) CD8+ T cells. Lines represent median values and each point is the mouse recipient of a unique stool donor. Statistical analyses were performed using Mann-Whitney tests. (TIF) [file ppat.1007611.s006.tif]

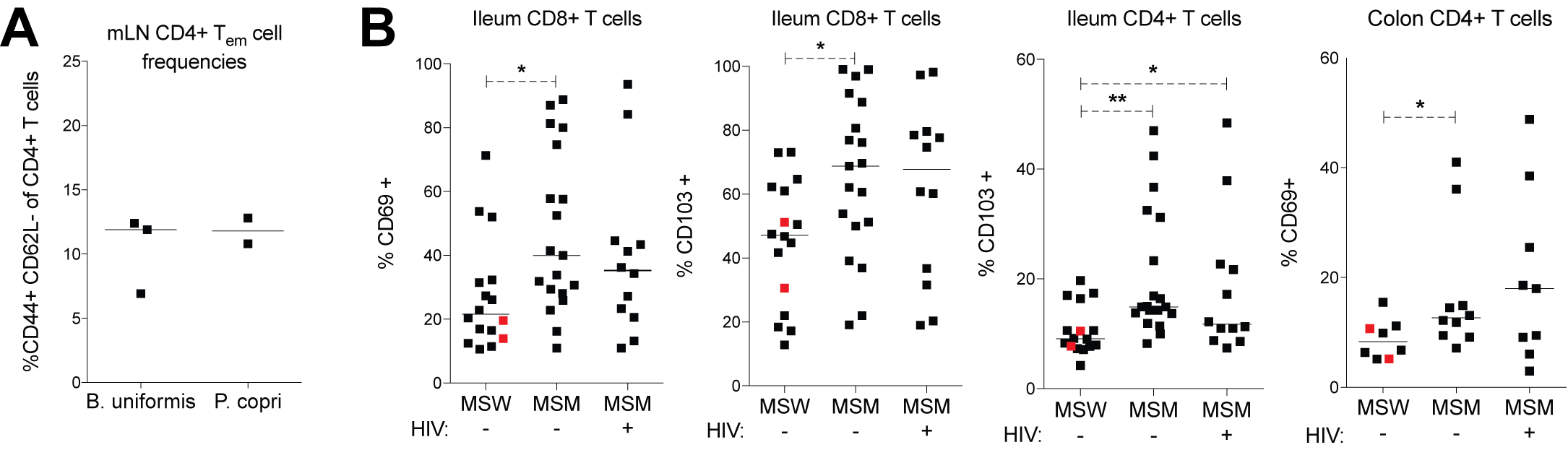

Supplement: S7 Fig — (A) Mice were monocolonized with either Bacteroides uniformis or Prevotella copri, and frequencies of CD4+ Tem cells in mesenteric lymph nodes were assessed at 21 days post-gavage. (B) Immune data from Fig 3 was modified to distinguish MSW stool recipients successfully colonized by Prevotella (red squares). Each data point represents a single mouse recipient of monoculture or stool. Lines represent medians. Statistical analyses were performed using t-tests to compare groups if data from both groups had normal distributions and Mann-Whitney tests to compare groups if data from at least one group had a non-parametric distribution. **** = p <0.0001, *** = p<0.001, ** = p<0.01, * = p<0.05. (TIF) [file ppat.1007611.s007.tif]

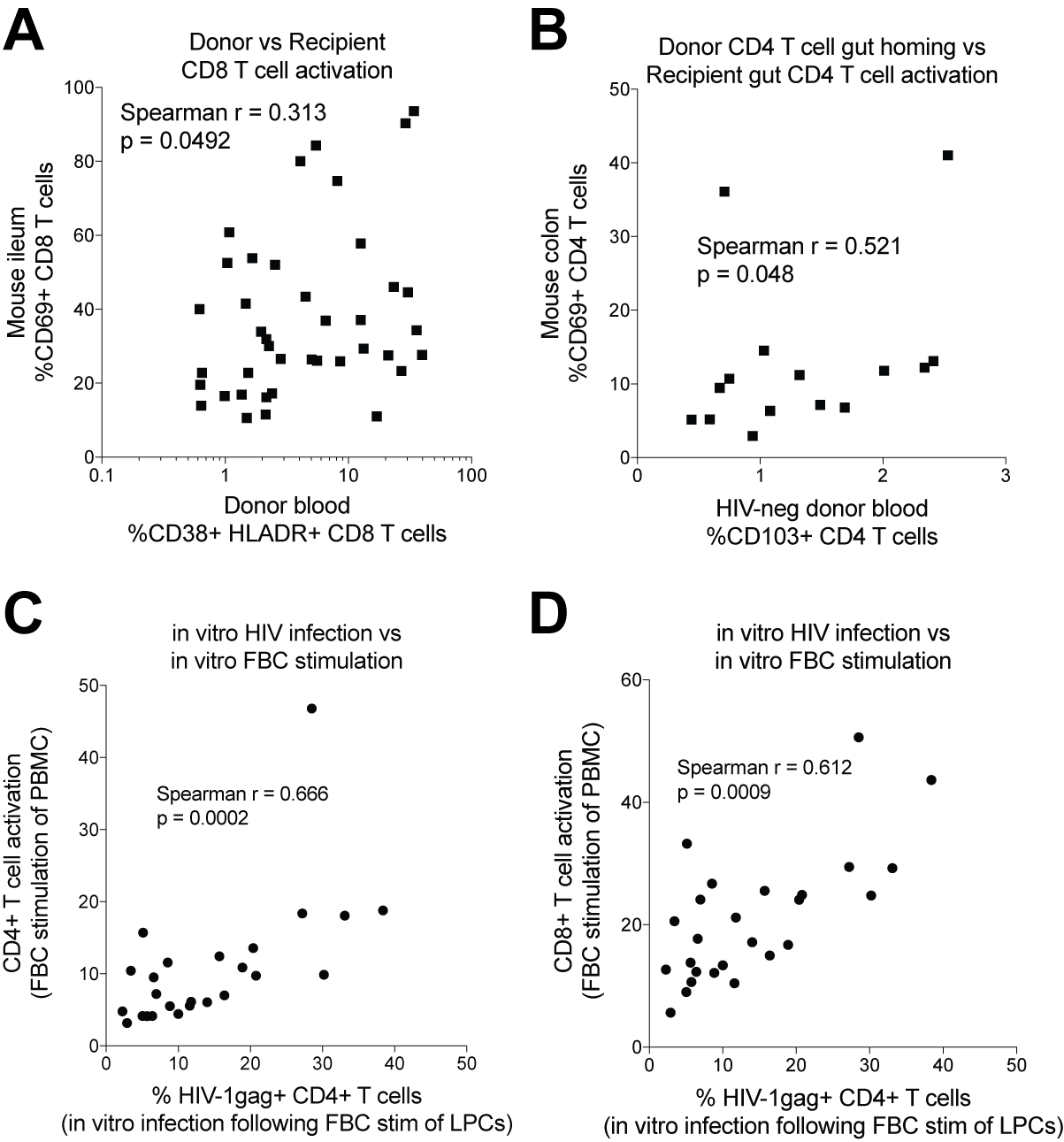

Supplement: S8 Fig — (A–B) Immune data from donors were correlated with immune data from mouse recipients. (A) Frequencies of blood CD38+ HLADR+ CD8+ T cells in all donors (plotted in log scale but correlated using linear data) were correlated with frequencies of ileum CD69+ CD8+ T cells in their recipients, and (B) frequencies of blood CD103+ CD4+ T cells in HIV-negative donors were correlated with frequencies of colon CD69+ CD4+ T cells in their recipients. Each data point represents a donor-recipient pair, with representative recipients used for donors tested in replicates. (C–D) In vitro infection data were correlated with previously published data [8] of in vitro FBC stimulation of primary human PBMC. HIV infection levels in LPCs stimulated by FBCs correlated with CD4+ T cell (A) and CD8+ T cell activation (B) in PBMC stimulated with the same FBCs. Each point represents an individual FBC that was used in both the infection assay in this study, and in previously published in vitro PBMC stimulations. Spearman r values and p values are shown. (TIF) [file ppat.1007611.s008.tif]

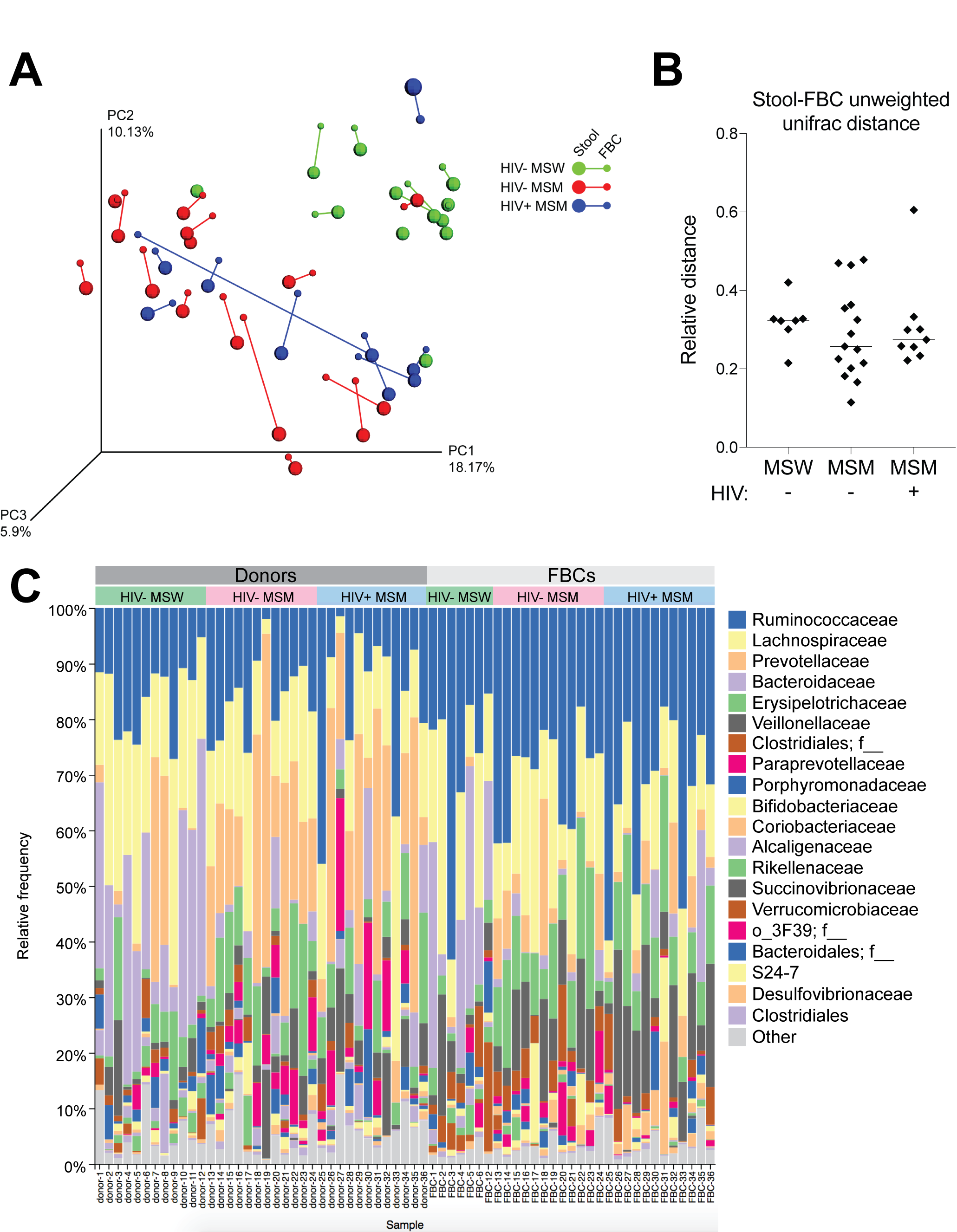

Supplement: S9 Fig — 16S rRNA gene sequencing was used to characterize the bacterial composition of original stool samples used for bacterial isolation, and fecal microbial communities (FBCs) following bacterial isolation. Sequencing data for five FBCs were not of high enough quality to be included in the final analyses. (A) Unweighted UniFrac clustering of stool (large dots) and FBC (small dots) compositions, with a line connecting each FBC to their original stool. Green dots are HIV-negative MSW, red dots are HIV-negative MSM, and blue dots are HIV-positive (ART-naïve) MSM. (B) Unweighted UniFrac distance between each FBC and their original stool were calculated and compared across groups. Each data point represents relative distance between an individual stool-FBC pair. Statistical analyses were performed using t-tests. No statistically significant differences were detected between groups. (C) Taxa bar chart showing the relative abundance of bacterial families in FBCs and original stools. Legend identifies the top 20 most abundant families across all samples. f__ = unclassified bacterial family. (TIF) [file ppat.1007611.s009.tif]
